# Effect of Ga_2_O_3_ Content on the Activity of Al_2_O_3_-Supported Catalysts for the CO_2_-Assisted Oxidative Dehydrogenation of Propane

**DOI:** 10.3390/nano15131029

**Published:** 2025-07-02

**Authors:** Alexandra Florou, Georgios Bampos, Panagiota D. Natsi, Aliki Kokka, Paraskevi Panagiotopoulou

**Affiliations:** 1 Laboratory of Environmental Catalysis, School of Chemical and Environmental Engineering, Technical University of Crete, GR-73100 Chania, Greece; aflorou@tuc.gr (A.F.); akokka@tuc.gr (A.K.); 2 Department of Chemical Engineering, University of Patras, GR-26504 Patras, Greece; geoba@chemeng.upatras.gr (G.B.); natsi@chemeng.upatras.gr (P.D.N.); 3 Institute of Geoenergy, Foundation for Research and Technology-Hellas (IG/FORTH), GR-73100 Chania, Greece

**Keywords:** CO_2_-assisted oxidative dehydrogenation of propane, propylene production, surface basicity/acidity, Ga_2_O_3_-Al_2_O_3_ catalysts, Ga_2_O_3_ loading, reaction mechanism, DRIFTS studies

## Abstract

Propylene production through the CO_2_-assisted oxidative dehydrogenation of propane (CO_2_-ODP) is an effective route able to address the ever-increasing demand for propylene and simultaneously utilize CO_2_. In this study, a series of alumina-supported gallium oxide catalysts of variable Ga_2_O_3_ loading was synthesized, characterized, and evaluated with respect to their activity for the CO_2_-ODP reaction. It was found that both the catalysts’ physicochemical characteristics and performance were strongly affected by the amount of Ga_2_O_3_ dispersed on Al_2_O_3_. Surface basicity was maximized for the sample containing 20 wt.% Ga_2_O_3_, whereas surface acidity was monotonically increased with increasing Ga_2_O_3_ loading. A volcano-type correlation was found between catalytic performance and acid/base properties, according to which propane conversion and propylene yield exhibited optimum values for intermediate surface basicity and acidity, which both correspond to the sample containing 30 wt.% Ga_2_O_3_. The dispersion of a suitable amount of Ga_2_O_3_ on the Al_2_O_3_ surface not only enhances the conversion of propane to propylene but also suppresses the formation of side products (C_2_H_4_, CH_4_, and C_2_H_6_) at temperatures of practical interest. The 30%Ga_2_O_3_-Al_2_O_3_ catalyst exhibited very good stability at 550 °C, where byproduct formation and carbon deposition were limited. Mechanistic studies indicated that the reaction proceeds through a two-step oxidative route with the participation of CO_2_ in the abstraction of H_2_, originating from propane dehydrogenation, through the reverse water–gas reaction (RWGS) reaction, shifting the thermodynamic equilibrium towards propylene generation.

## 1. Introduction

The efficient production of propylene (C_3_H_6_), one of the most important building blocks of the chemical industry, has become attractive during the last decade in order to address the rapid growth of the C_3_H_6_ market [1,2,3,4,5]. Propylene is conventionally produced as a byproduct either via the hydrocarbons steam cracking process used to produce ethylene or the catalytic cracking of hydrocarbons used in refineries to produce gasoline. However, both these technologies (a) are energy-intensive, leading to lower propylene selectivity compared to ethylene, (b) require product separation processes that are energy and financially costly, and (c) result in increased greenhouse gas emissions [1,6]. Therefore, nowadays, the development of cost-effective and environmentally friendly technologies for the on-purpose propylene synthesis is vital, attempting to shorten the already wide gap between industrial production and existing needs [1,3,7]. In this respect, the dehydrogenation of propane to propylene is an attractive route, taking into account the abundant availability of propane from shale gas [1,3,7]. However, the high endothermicity of this reaction requires high temperatures in order to be operable, favoring the undesirable propane and/or propylene decomposition, yielding lighter hydrocarbons and coke, which are responsible for catalyst deactivation and low propylene yields.

An alternative and promising approach for this purpose is the oxidative dehydrogenation of propane using a soft oxidant like CO_2_ (CO_2_-ODP) (1), which is exothermic and thus operable at relatively low temperatures and capable of overcoming the drawbacks of the propane dehydrogenation process [4,8,9]. In addition to propylene production, this reaction has a significant environmental impact as it utilizes CO_2_ emissions, thus mitigating the greenhouse effect. Moreover, the presence of CO_2_ (a) shifts the equilibrium towards propylene formation by consuming the produced hydrogen via the reverse water–gas reaction (RWGS) (2) and (b) enables coke removal from the catalyst surface by converting it to CO via the reverse Boudouard reaction (3) [3,4].

(1) $${\mathrm{CO}}_{2}+\;C_{3}H_{8}\;↔\;C_{3}H_{6}+\;\mathrm{CO}\;+\;H_{2}O\;{\Delta H}_{298Κ}^{0}=165.4\;\mathrm{kJ}/\mathrm{mol}$$

(2) $${\mathrm{CO}}_{2}+\;H_{2}\;↔\;\mathrm{CO}\;+\;H_{2}O\;{\Delta H}_{298Κ}^{0}=41.1\;\mathrm{kJ}/\mathrm{mol}$$

(3) $${\mathrm{CO}}_{2}+\;C\;↔\;2\mathrm{CO}\;{\Delta H}_{298Κ}^{0}=172.4\;\mathrm{kJ}/\mathrm{mol}$$

It should be noted, however, that, under certain conditions, the reactions of C_3_H_8_ hydrogenolysis and C_3_H_8_/C_3_H_6_ decomposition, which are responsible for coking of catalysts and low propylene yields, may not be avoided and prevail against the CO_2_-ODP and reverse Boudouard reactions [4]. Therefore, active catalysts able to activate the thermodynamically stable CO_2_ and selectively convert propane to propylene at moderate temperatures, suppressing side reactions, should be developed. Previous studies have shown that the appropriate selection of the active phase, the support, the catalyst synthesis method, and the pretreatment conditions, as well as the modification of catalysts by the suitable amount and type of metal additives, are crucial factors in catalyst development for the CO_2_-ODP process [4,5,7,8,10].

Two general types of catalysts commonly studied for the CO_2_-ODP process are gallium-based and chromium-based catalysts. Although chromium-based catalysts exhibit high catalytic activity [11,12], they suffer from chromium toxicity, making them unsuitable for the production of propylene [9]. Contrary, gallium is less toxic than chromium, particularly compared to hexavalent chromium (Cr^6+^), which is carcinogenic and environmentally hazardous, whereas using gallium-based catalysts also reduces environmental and health risks during synthesis, handling, and disposal. Concerning the cost, although gallium catalysts are more expensive in terms of raw materials and initial investment, they may offer economic advantages in selectivity, stability, and environmental compliance. On the other hand, chromium-based catalysts are cheaper and more accessible, but come with hidden costs related to toxicity, regulation, and shorter operational lifetime.

Supported Ga_2_O_3_ catalysts have been reported to be effective for the dehydrogenation of light alkanes either under non-oxidative or oxidative reaction conditions, with their activity generally dependent on the number and type of acid/base sites, the oxidation state and coordination environment of gallium, the nature of the support as well as the synthesis method used. Gashoul Daresibi et al. [2] reported that the higher surface moderate acidity, the larger fraction of Ga-O-Al linkages and the higher dispersion of Ga_2_O_3_ on Al_2_O_3_ support prepared by the atomic layer deposition method were responsible for the higher propane conversion (38% at 600 °C) and propylene selectivity (82% at 600 °C) compared to those prepared by the impregnation method. Low-coordinate Ga^3+^ alkyl and hydride species were found to be active intermediates able to activate the C–H bond under propane dehydrogenation conditions over Ga–SiO_2_ and Ga–H-BEA catalysts, providing evidence that the coordination environment of gallium is important [13]. Concerning the oxidation state of gallium oxide on the surface of various supports (Al_2_O_3_, HZSM-5, SiO_2_, H-BEA), controversial results have been reported, dependent mainly on the nature of the support. For example, Ga 2p_3/2_ or Ga 3d peaks centered around binding energies typical of Ga_2_O_3_ were observed in XPS spectra obtained from Ga_2_O_3_-Al_2_O_3_, Ga_2_O_3_-HZSM-5 and Ga_2_O_3_-SiO_2_ catalysts indicating that Ga mainly exists in the +3 oxidation state (Ga^3+^) even after exposure to propane dehydrogenation conditions in the presence of CO_2_ [14,15,16]. On the other hand, Shao et al. [17] demonstrated by XPS conducted over 5%Ga_2_O_3_/ZSM-5 and 5%Ga_2_O_3_/Al_2_O_3_ catalysts the presence of both Ga^3+^ and Ga^δ+^ species (δ < 2), suggesting that Ga_2_O_3_ can be partially reduced during the catalyst synthesis process. The investigation of the reducibility of Ga_2_O_3_-based catalysts has been the subject of many studies since it has been found to be correlated with the role of CO_2_, influencing the reaction pathway [13,18].

Tedeeva et al. [19] studied the effect of the support nature over Ga catalysts dispersed on various types of silicon dioxides and demonstrated that higher catalytic activity can be achieved over catalysts characterized by high Ga_2_O_3_ dispersion and low acidity. On the contrary, many researchers agree that the higher number of acid sites decreases the activation energy barrier for the C–H bond activation, facilitating the conversion of propane to propylene [2,20]. In our previous studies, it was found that catalytic activity for the CO_2_-ODP reaction over composite metal oxides is highly dependent on the type of active face and the support and is determined by the number and strength of both basic and acidic sites on the catalyst surface [4,5,8]. Specifically, it was demonstrated that propane conversion and propylene yield can be significantly improved by the addition of 10 wt.% metal oxides (e.g., ZrO_2_, CeO_2_, CaO, Cr_2_O_3_, Ga_2_O_3_, SnO_2_) on TiO_2_ or SiO_2_ surface, with the catalysts containing Ga_2_O_3_ or Cr_2_O_3_ exhibiting superior activity [4,5]. In the case of titania-supported catalysts, the moderate surface basicity, the high surface acidity, the increased reducibility, and the decrease in TiO_2_ primary crystallite size were found to be responsible for the improved performance of Ga_2_O_3_-TiO_2_ and Cr_2_O_3_-TiO_2_ [4]. In the case of silica-supported catalysts, both a moderate surface acidity and basicity seem to be desirable for the selective conversion of C_3_H_8_ towards C_3_H_6_ and the suppression of side reactions yielding C_2_H_4_, CH_4,_ and coke [5]. Concerning the effect of the support nature, it was found that catalytic performance was higher when Ga_2_O_3_ was dispersed on Al_2_O_3_, which was characterized by the highest acid site density and a moderate basicity compared to TiO_2_ or SiO_2_ [8].

Although the CO_2_-ODP reaction has been widely studied with respect to the nature of the active face and the support, only a few studies have been reported thus far regarding the active face content. Gashoul Daresibi et al. [2] reported that catalytic performance can be significantly improved with increasing Ga loading from 1 to 2.9%. Tedeeva et al. [19] investigated the effect of Ga content on various types of SiO_2_ support in a wider range of 3–50% and found that the best results can be achieved for the sample containing 7%Ga dispersed on the surface of a SiO_2_ sample, which was characterized by the highest specific surface area thereby enabling a high Ga dispersion. Moreover, Han et al. [3] synthesized Ga_2_O_3_-Al_2_O_3_ nanofibers of various Ga/Al molar ratios of 1:8, 1:4, 3:8, and 1:2 employing the electrospinning method, and demonstrated that propane conversion and propylene selectivity were optimized for Ga/Al = 3:8, taking values of 48.4% and 96.8%, respectively.

The present study deals with the synthesis and characterization of Ga_2_O_3_-Al_2_O_3_ catalysts of various Ga_2_O_3_ concentrations (0–40 wt.%) as well as their evaluation with respect to their performance for the CO_2_-ODP reaction. The aim is to determine the influence of Ga_2_O_3_ content on the physicochemical characteristics of catalysts and identify its correlation with propane conversion and propylene selectivity in order to optimize the process efficiency. Mechanistic aspects of the CO_2_-ODP reaction over selected catalysts were also investigated employing in situ FTIR and transient mass spectrometry (transient-MS) techniques. Among others, the new findings of the present study include: (a) Remarkable volcano type correlations between the Ga_2_O_3_ content and the physicochemical properties, the catalytic activity as well as the propylene selectivity and yield, which enable the optimization of the modifier concentration that is able to result in superior efficiency for the CO_2_-ODP reaction; (b) the development of a highly active catalyst capable to achieve higher propane conversions and propylene yields compared to those reported in previous studies; (c) The optimization of reaction conditions that are able to suppress side reactions and thus, control the carbon deposition rate, leading to satisfactory stable performance; (d) The identification of the nature of adsorbed surface species involved in the propane dehydrogenation to propylene and their relation with the gas phase products, which enabled us to propose a possible reaction pathway over Ga_2_O_3_-Al_2_O_3_ catalysts.

## 2. Materials and Methods

### 2.1. Synthesis of Catalysts

The incipient wetness impregnation method was used for the synthesis of x% Ga_2_O_3_-Al_2_O_3_ (x: 0, 10, 20, 30, 40 wt.%) catalysts. A commercial Al_2_O_3_ (Alfa Aesar, Kandel, Germany) was used as a carrier, which was impregnated with the appropriate amount of Ga(NO_3_)_3_·6H_2_O (Sigma Aldrich, Darmstadt, Germany) precursor to achieve the desired gallium oxide content. The procedure involved progressive heating of the resulting suspension at 80 °C under magnetic stirring until water evaporation, drying the samples at 110 °C for 12 h, and calcination in an air atmosphere using a heating rate of 3 °C/min up to 600 °C, where it remained for 3 h to obtain the final catalysts. For comparison purposes, the bare Al_2_O_3_ powder as well as a commercially available Ga_2_O_3_ (Thermo Scientific, Waltham, MA, USA) powder were also treated following the same procedure.

### 2.2. Characterization of Catalysts

The specific surface area of catalysts was calculated according to the Brunauer–Emmett–Teller (BET) method, and the pore volume and size according to the Barrett–Joyner–Halenda (BJH) method, following measurements of nitrogen adsorption at −196 °C using a Quantachrome gas sorption unit (Quantachrome Instruments, Boynton Beach, FL, USA). Prior to these experiments, the samples were dried at 110 °C for 2 h. X-ray diffraction (XRD) patterns of the synthesized catalysts were obtained with a Bruker D8 Advance (Billerica, MA, USA) diffractometer (CuKα radiation). The samples were scanned in the 2θ range of 20–80° at a rate of 0.05 °/s.

Scanning Electron Microscopy (SEM) was carried out using a JEOL JSM 6300 microscope (Akishima, Tokyo, Japan) equipped with an energy dispersive spectrometer (EDS, ISIS Link 300, Oxford Instruments, Oxford, UK) for elemental analysis. Transmission Electron Microscopy (TEM) images were also collected from selected catalysts using a JEOL JEM-2100 instrument (JEOL, Tokyo, Japan), which operates at 200 kV (point resolution 0.23 nm) with the use of an Erlangshen CCD Camera (Gatan Model 782 ES500W, Pleasanton, CA, USA).

CO_2_-temperature programmed desorption (CO_2_-TPD) experiments were performed to determine the surface basicity of Ga_2_O_3_-Al_2_O_3_ catalysts using a mass spectrometer (Pfeiffer Vacuum, Asslar, Germany) for the online analysis of gases at the effluent of a quartz fixed-bed reactor where the catalyst was placed. The sample (0.15 g) was initially treated at 450 °C by flowing He (40 cm^3^ min^−1^) for 15 min in order to remove water or other impurities from the catalyst surface. The treated catalyst was then cooled down and exposed for 30 min to a gas stream of 5% CO_2_/He (40 cm^3^ min^−1^) through a flow system which was directly connected to the reactor inlet. The flow was subsequently changed to He for 30 min to remove the physisorbed CO_2,_ and the TPD was started by raising the sample temperature from 25 to 750 °C with a heating rate of 10 °C/min. The desorption of CO_2_ and/or possibly CO was continuously monitored by recording the transient-MS signals at *m*/*z* = 44 (CO_2_) and 28 (CO).

The adsorption/desorption characteristics of CO_2_ on the surface of Ga_2_O_3_-Al_2_O_3_ catalysts were also studied employing in situ diffuse reflectance infrared Fourier transform spectroscopy (DRIFTS). The FTIR spectra (resolution 4 cm^−1^) were recorded on a Nicolet iS20 (Thermo Fischer Scientific, Waltham, MA, USA) spectrometer equipped with a liquid nitrogen cooled MCT detector and a KBr beam splitter. Prior to CO_2_ adsorption, the catalyst placed in the DRIFTS cell was purged by helium (30 cm^3^ min^−1^) at 450 °C for 60 min. The temperature was then progressively decreased to 25 °C, collecting in parallel the background spectra at the desired temperatures. The flow was then changed to 5% CO_2_/He (30 cm^3^ min^−1^) for 30 min and subsequently to He for 10 min. The FTIR spectrum at 25 °C was then recorded, followed by a successive rise in temperature up to 450 °C. During this stage, similar FTIR spectra were collected at 100, 150, 200, 250, 300, 350, 400, and 450 °C, after a 3-min dwell period at each temperature.

The same FTIR spectrometer was used for the investigation of catalysts’ surface acidity employing pyridine adsorption/desorption experiments. The procedure involved ex situ adsorption of pyridine until saturation by suspending 0.06 g of dried catalyst in an aqueous solution containing 5% pyridine. After 2 h of continuous stirring, the suspension was filtered and subsequently dried at 60 °C for 1 h, aiming to remove water and/or weakly adsorbed pyridine. The catalyst was then placed in the diffuse reflectance cell, and the first spectrum was collected at 25 °C in He flow, followed by a successive increase in temperature up to 500 °C, where infrared spectra were recorded at certain temperatures after 3 min of retention at each. Background spectra were also recorded at the same temperatures under a helium atmosphere over dried catalyst and subtracted from those obtained following the adsorption of pyridine.

The surface acidity of Ga_2_O_3_-Al_2_O_3_ catalysts was also investigated employing a potentiometric titration method according to which all acid sites can be determined by using a strong base as a titrant [21,22,23,24,25]. Titrations were carried out in an automatic titration system (lab-made) that consisted of a mechanically controlled computer-operated syringe. Based on the pH deviation from a set point value, the system allows the addition of acid or base standard solutions into the investigated suspended sample to achieve the desired pH value. Briefly, an amount of 0.03 g of dried catalyst was suspended in a KNO_3_ 0.05 M solution and remained under stirring at 25 °C for 30 min in order to achieve catalyst concentrations of 2 g/L. The pH was adjusted to 2.5–3 with HCl 0.1 M. Titrations were then conducted with a standard solution of NaOH 0.1 M, until pH 11.7.

The titration results were analyzed by the Gran method, which allows the estimation of the concentration of surface acidic sites [21,22,23,24]. The Gran’s function was defined as follows:

(4) $$G\left(v\right)=\left(V_{o}+V\right)\cdot 10^{-pH}\;\mathrm{at}\;\mathrm{pH}\;<7$$

(5) $$G\left(v\right)=\left(V_{o}+V\right)\cdot 10^{\left(pH-14\right)}\;\mathrm{at}\;\mathrm{pH}\;>7$$

where *V_o_* represents the initial sample volume (cm^3^) and *V* is the volume of added titrant (cm^3^) [23]. Based on this method, the typical sigmoidal titration curve is transformed into a linear one by plotting the calculated values of *G*(*V*) = *V*·[H^+^] as a function of the titration volume (*V*) of the added strong base [22,24,26]. As a result, a straight line is obtained, which intersects the volume axis at the equivalence volume (V_eq_). The V_eq_ value, combined with the strong base concentration value (C_OH_), is used for estimating the titrated acid sites. The slope of the line corresponds to the protonation constant (K) of the specific acid site. In cases where the titrated material contains multiple acid sites of variable strength, the transformed curve will consist of several linear sections, each corresponding to a distinct acid site with its own protonation constant. Curvature may appear at the transitions between sections due to overlapping protonation equilibria.

Based on the above, the total acidity of catalysts can be divided into three distinct types of acidic sites of different strengths, referred to as strong, weak, and very weak acid sites [23]. The corresponding surface acidities (in mmol g^−1^) can be estimated using the following equations:

For the strong acid sites (at pH < 4):

(6) $$A_{s}=\frac{\left(V_{a}-V_{aN}\right)}{m}\cdot N_{o}$$

For the weak acid sites (at 4 < pH < 7):

(7) $$A_{w}=\frac{\left(V_{e}-V_{a}\right)-\left(V_{eN}-V_{aN}\right)}{m}\cdot N_{o}$$

For the very weak acid sites (at pH > 7):

(8) $$A_{vw}=\frac{\left[\left(V_{b}-V_{e}\right)-\left(V_{bN}-V_{eN}\right)\right]\left[\left(V-V_{b}\right)-\left(V_{N}-V_{bN}\right)\right]}{m}\cdot N_{o}$$

where *N_o_* represents the normality of the base used (NaOH 0.1 M), *V* (mL) and *V_N_* (mL) correspond to the total volume of the base added after titration of the catalyst and the volume of the control sample (in the absence of catalyst), respectively, *V_e_* (mL) is referred to the volume of the base in the end-point of titration and *m* (g) is the mass of the sample. Moreover, *V_a_* and *V_aN_* represent the acid equivalence points (for pH < 7) estimated by the points that the equation (4) intersects the x-axis for the catalyst and control sample, respectively, whereas *V_b_* and *V_bN_* represent the basic equivalence points (for pH > 7) estimated by the points that the equation (7) intersects the x-axis for the catalyst and the control sample, respectively. Therefore, the *(V_a_* − *V_aN_)* corresponds to the titrant volume necessary for the reaction with strong acid sites at pH < 4, the (*V_e_* − *V_a_*) and (*V_eN_* − *V_aN_*) are referred to the ionization of surface sites at 4 < pH < 7 for the catalyst and the control sample, respectively, and (*V_b_* − *V_e_*) and (*V_bN_* − *V_eN_*) represent the excess base consumption for the catalyst and the control sample, respectively, which is usually attributed to the interaction of basic functional groups (like OH groups) with H^+^ ions at pH > 7. The total acidity of catalysts can be calculated as the sum of A_s_, A_w_ and A_vw_.

The reducibility of selected catalysts was investigated by temperature-programmed reduction with H_2_ (H_2_-TPR) using the mass spectrometer described above. In these experiments, the catalyst was initially oxidized in 5%O_2_/He flow at 500 °C for 30 min, followed by cooling at 25 °C in He and subsequently exposure to 5%H_2_/He. After maintaining the catalyst at 25 °C for 10 min, the TPR experiment was initiated using a heating rate of 10 °C/min.

### 2.3. Catalytic Performance Tests

The performance of catalysts for the CO_2_-ODP reaction was evaluated at atmospheric pressure in the temperature range of 450–750 °C using an experimental setup consisting of a quartz fixed-bed reactor with an inner diameter of 4 mm and 45 cm length connected with a gas chromatograph (Shimadzu 2014, Kyoto, Japan) equipped with FID and TCD detectors for the analysis of the effluent gas. The mass of catalyst used was 0.5 g and placed in an expanded section with dimensions of 10 mm inner diameter × 5 cm length in the middle of the reactor. The reaction mixture consisted of 5% C_3_H_8_ and 25% CO_2_, balanced with He, and was fed to the reactor with a flow rate of 50 mL min^−1^. Prior to catalytic performance tests, the catalyst was pretreated in a stream of helium at 450 °C for 1 h. The concentration of reactants and products was measured following a progressive increase in temperature from 450 to 750 °C after remaining at each temperature for 30 min to reach steady state. Time on stream (TOS) stability tests were also carried out at a constant temperature using the same pretreatment and reaction conditions. More details about the experimental setup and procedure employed can be found in our recent publications [4,5,8].

The equations used for the estimation of the C_3_H_8_ conversion ($X_{C_{3}H_{8}}$), C_3_H_6_ yield ($Y_{C_{3}H_{6}}$) and selectivity towards each product (*S*_Cn_) are described below:

(9) $$X_{C_{3}H_{8}}=\frac{{\left[C_{3}H_{8}\right]}_{in}\cdot F_{in}-{\left[C_{3}H_{8}\right]}_{out}\cdot F_{out}}{{\left[C_{3}H_{8}\right]}_{in}\cdot F_{in}}\times 100$$

(10) $$Y_{C_{3}H_{6}}=(X_{C_{3}H_{8}}\cdot S_{C_{3}H_{6}})/100$$

(11) $$S_{C_{n}}=\frac{\left[C_{n}\right]\cdot n}{\left[CO\right]+\left[CH_{4}\right]+2\cdot \left(\left[C_{2}H_{4}\right]+\left[C_{2}H_{6}\right]\right)+3\cdot \left(\left[C_{3}H_{6}\right]\right)}\times 100$$

where *F_in_* and *F_out_* correspond to the inlet and outlet molar flow rate, [C_3_H_8_]_in_ and [C_3_H_8_]_out_ denote the *v*/*v* concentrations of C_3_H_8_ in the inlet and outlet of the reactor, respectively, [C_n_] the *v*/*v* concentration of each product component (i.e., C_3_H_6_, C_2_H_4_, C_2_H_6_, CH_4_ and CO), and *n* denotes the carbon atoms number of each molecule (e.g., 1 for CO and CH_4_, 2 for C_2_H_4_ and C_2_H_6_, 3 for C_3_H_6_).

### 2.4. In Situ FTIR Spectroscopy Under Reaction Conditions

Experiments of in situ *FTIR* spectroscopy were also carried out under conditions of CO_2_-assisted oxidative dehydrogenation of propane using the FTIR spectrometer described above. The catalyst was placed in the DRIFT cell and initially treated at 500 °C for 30 min with a flow of helium (30 cm^3^ min^−1^). The temperature was then decreased to 25 °C under the same atmosphere, followed by exposure of the catalyst to a reaction mixture of 1% C_3_H_8_ + 5% CO_2_ (in He) (30 cm^3^ min^−1^). The first infrared spectrum was recorded after 15 min on stream. A progressive increase in temperature then took place up to 500 °C under the flow of the reactant mixture. During this stage, certain spectra were collected at selected temperatures after a 15-min stay at each of them. Similar spectra were recorded under helium, which were used as backgrounds to normalize those obtained under reaction conditions.

### 2.5. Temperature-Programmed Surface Reaction (TPSR) Experiments with Mass Spectrometry

The TPSR experiments were performed at atmospheric pressure in a quartz fixed-bed reactor with a catalyst loading of 0.5 g. The catalyst was pretreated in a stream of He (40 cm^3^ min^−1^) at 450 °C for 15 min and subsequently was cooled down to 25 °C under the same atmosphere. The flow was then changed to 1% C_3_H_8_ + 5% CO_2_ (in He). The catalyst was kept at 25 °C for 15 min, followed by a linear heating (*β* = 10 °C min^−1^) to 750 °C. The effluent gas composition was online monitored using the mass spectrometer described above by recording the transient-MS signals at *m*/*z* = 2 (H_2_), 15 (CH_4_), 18 (H_2_O), 28 (CO), 29 (C_3_H_8_), 41 (C_3_H_6_), 27 (C_2_H_4_), 30 (C_2_H_6_) and 44 (CO_2_). The MS responses were calibrated using gas mixtures of known composition. In certain cases (e.g., CO_2_-CO, C_3_H_8_-C_2_H_4_ signals), the cracking coefficient was also considered in estimating the concentration of gases in the reactor outlet.

### 2.6. Temperature-Programmed Oxidation (TPO) Experiments with Mass Spectrometry

After completion of the TOS stability tests and the TPSR experiments, the flow was changed to He, and the temperature was decreased to 25 °C, where the catalyst was exposed to a 1%O_2_ (in He) stream (40 cm^3^ min^−1^). A temperature-programmed oxidation was initiated after 10 min by increasing the temperature from 25 to 800 °C at a rate of 10 °C/min. The CO_2_ and/or CO produced via carbon oxidation were continuously monitored by recording the transient-MS signals at *m*/*z* = 44 (CO_2_) and 28 (CO) using the mass spectrometer described above.

## 3. Results and Discussion

### 3.1. Catalyst Characterization

Results of N_2_ adsorption experiments are summarized in Table 1, where a progressive decrease in the specific surface area can be observed from 64.4 to 46.2 m^2^/g as the gallium oxide loading increased from 0 to 40 wt.%, most possibly due to a partial blocking of alumina pores induced by Ga_2_O_3_ addition [2,4,5,27]. The BET surface area measured for the bare Ga_2_O_3_ powder was significantly lower (4 m^2^/g). The pore volume and the mean pore diameter were measured over bare Al_2_O_3_, 10%Ga_2_O_3_-Al_2_O_3_ and 30%Ga_2_O_3_-Al_2_O_3_ and found to progressively decrease with increasing Ga_2_O_3_ content, taking values of 0.187, 0.166 and 0.145 cm^3^ g^−1^, and 8.3, 7.9 and 7.5 nm, respectively.

**Figure 1:**
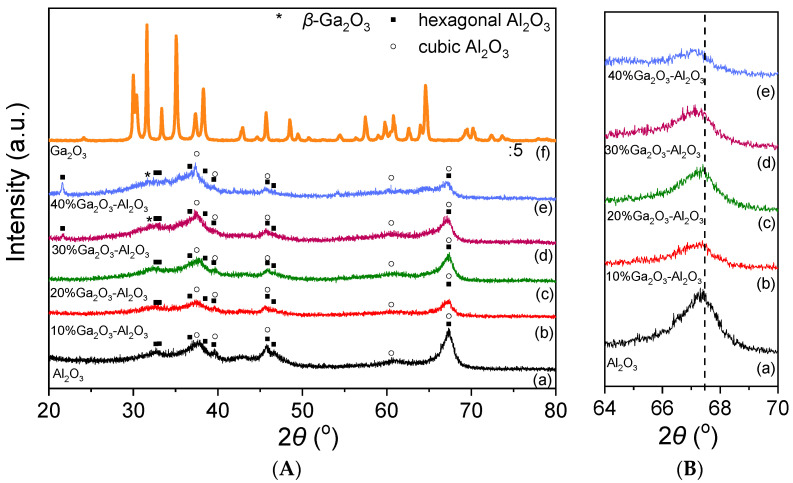
(**A**) X-ray diffraction patterns obtained from Al_2_O_3_, Ga_2_O_3,_ and x%Ga_2_O_3_-Al_2_O_3_ catalysts. (**B**) Magnification of Al_2_O_3_ and x%Ga_2_O_3_-Al_2_O_3_ diffractograms in the region 64° < 2*θ* < 70°.

The X-ray diffractograms obtained from the investigated catalysts are illustrated in Figure 1A. In the case of Al_2_O_3_ and Ga_2_O_3_-Al_2_O_3_ catalysts, the typical diffraction peaks of the hexagonal and cubic Al_2_O_3_ structure were detected. Specifically, XRD peaks located at 2θ values equal to 33.07°, 36.69°, 38.51°, 39.52°, 45.87°, 46.62° and 67.4° correspond to (006), (212), (205), (300), (304), (221) and (414) planes of hexagonal Al_2_O_3_ (JCPDS Card No. 21-10), respectively, whereas diffraction peaks located at 37.8°, 39.67°, 45.90°, 60.53° and 67.34° diffraction angles correspond to (311), (222), (400), (511) and (440) Miller indices of cubic Al_2_O_3_ (JCPDS Card No. 4-880), respectively. An extra peak centered at 21.63° was detected only for the 30%Ga_2_O_3_-Al_2_O_3_ and 40%Ga_2_O_3_-Al_2_O_3_ samples and can be attributed to the (004) phase of hexagonal alumina (JCPDS Card No. 21-10). It is of interest to note that the position of the diffraction angle assigned to the (440) plane of cubic alumina was slightly shifted towards lower angles as the Ga_2_O_3_ content was becoming higher (Figure 1B). A similar shift was previously attributed to the formation of a solid solution induced by the incorporation of Ga^3+^ ions of a higher ionic radius than that of Al^3+^ ions into the Al_2_O_3_ structure [2,3,14,28,29].

No diffraction peaks of gallium oxide were discerned for the samples containing 10 and 20 wt.% Ga_2_O_3,_ suggesting that either Ga_2_O_3_ particles were highly dispersed on the Al_2_O_3_ surface or Ga_2_O_3_ was amorphous or a single-phase oxide in Al_2_O_3_. However, an additional peak located at 2θ = 31.6° assigned to the (002) plane of *β*-Ga_2_O_3_ can be discerned in the XRD pattern of the 30% and 40%Ga_2_O_3_-Al_2_O_3_ catalysts [30]. More peaks corresponding to gallium oxide may also coexist in the diffractograms but cannot be discerned due to overlapping with alumina peaks. Regarding the XRD pattern obtained from the bare Ga_2_O_3_, it was found to consist of peaks attributed to *β*-Ga_2_O_3_ (JCPDS Card No. 41-1103).

The morphology and the elements distribution of a selected catalyst, specifically, the 10%Ga_2_O_3_-Al_2_O_3_, was investigated with SEM and EDS analysis. A representative image, along with the element mapping of Ga and the EDS profile obtained, is presented in Figure S1. The EDS analysis confirmed the presence of Ga, O, and Al elements, while the elemental mapping demonstrated that Ga was homogeneously distributed on the surface of Al_2_O_3_. The weight percentage of Ga, Al, and O estimated by the EDS analysis was found to be equal to ca. 5.2 wt.%, 42.1 wt.%, and 52.7 wt.%, respectively. Representative TEM images and the selected area electron diffraction (SAED) patterns were obtained from bare Al_2_O_3_ and a selected Ga_2_O_3_-containing catalyst, the 10%Ga_2_O_3_-Al_2_O_3_ (Figure S2). It was found that both Al_2_O_3_ and 10%Ga_2_O_3_-Al_2_O_3_ catalysts consist of spherical Al_2_O_3_ nanoparticles with a diameter of about 6–8 nm. In the SAED spectra, the observed diffraction rings noted by spots 1, 2, 3, 4, 5, and 6 correspond to d-spacing values equal to 3, 2.4, 1.98, 1.63, 1.43, and 1.39 Å, respectively, of an unknown Al_2_O_3_ structure (JCPDS Card No. 2-1422). Taking into account that a cubic and hexagonal Al_2_O_3_ structure was identified in XRD measurements for both bare Al_2_O_3_ and 10%Ga_2_O_3_-Al_2_O_3_ samples, a polycrystalline structure of the Al_2_O_3_ used as support can be suggested [8]. No reflections attributed to Ga_2_O_3_ structure were detected over the 10%Ga_2_O_3_-Al_2_O_3_ sample, indicating that Ga_2_O_3_ particles were either well dispersed or amorphous. Results indicated that the morphology of alumina does not change with the addition of 10 wt.% Ga_2_O_3_.

**Figure 2:**
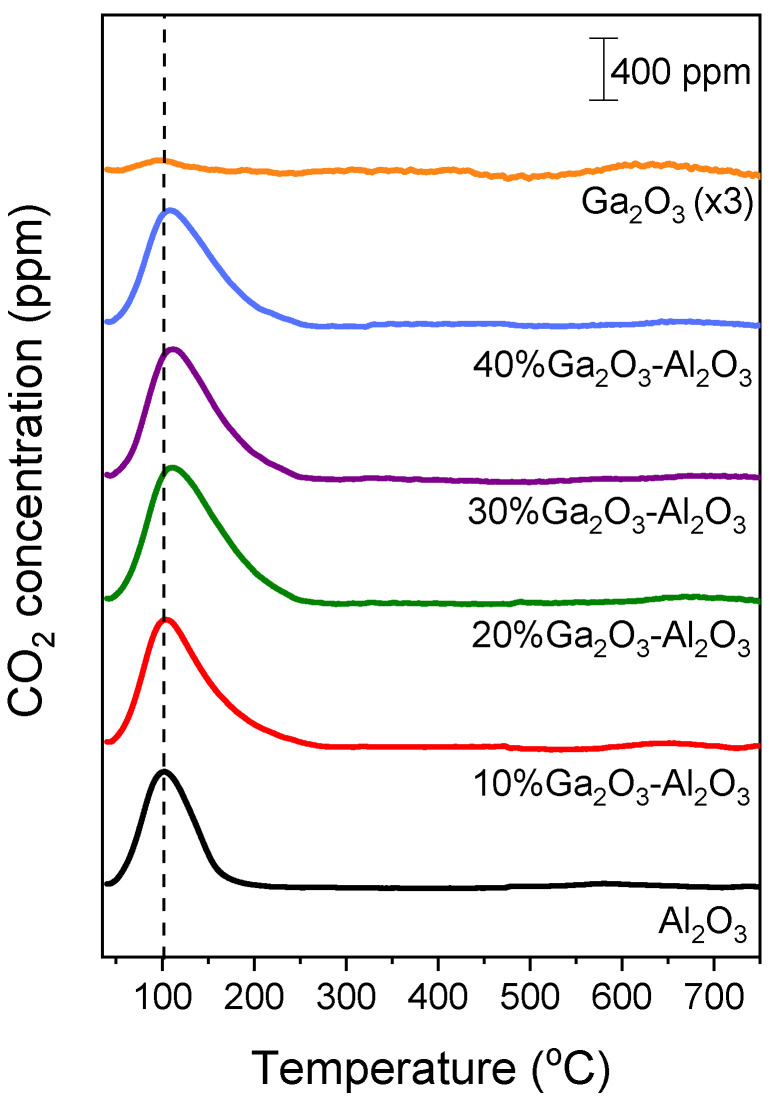
CO_2_-TPD profiles obtained from Al_2_O_3_, Ga_2_O_3_, and x%Ga_2_O_3_-Al_2_O_3_ catalysts.

**Figure 3:**
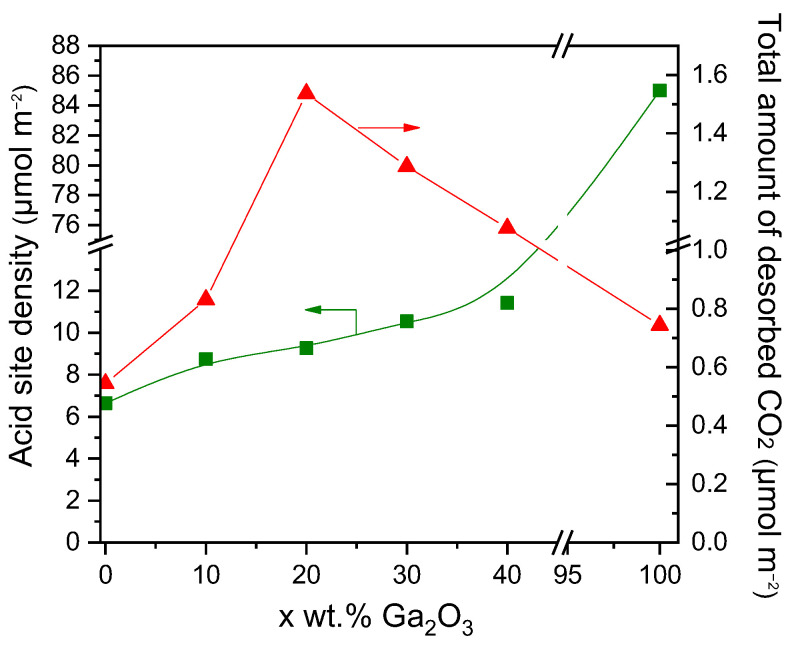
Effect of Ga_2_O_3_ content on the surface basicity estimated by CO_2_-TPD experiments and the acid site density estimated by the potentiometric titration experiments of the synthesized catalysts.

Results of CO_2_-TPD experiments obtained over the investigated catalysts employing the MS technique are presented in Figure 2, where the concentration of CO_2_ (in ppm) was plotted as a function of temperature for all catalysts examined. A low temperature (LT) desorption peak was observed over bare Al_2_O_3_ and x%Ga_2_O_3_-Al_2_O_3_ catalysts, which is related to CO_2_ desorption from weak basic sites [4,5,8,31,32]. The position of this peak was progressively shifted from 102 °C for bare Al_2_O_3_ to 111 °C for the samples containing 20, 30, and 40 wt.% Ga_2_O_3_ indicating that the strength of CO_2_ adsorption was enhanced in the presence of Ga_2_O_3_ in agreement with previous study [31]. A high temperature (HT) broad peak can hardly be discerned between 500 and 700 °C for all catalysts examined, which is associated with the desorption of CO_2_ from moderate/strong basic sites [4,5,8,32]. The intensity of both LT and HT peaks was too low for the bare Ga_2_O_3_ sample, most probably due to the low basicity in combination with the low specific surface area of this sample. The area below the LT and HT peaks was integrated to estimate the amount of CO_2_ (in μmol g^−1^) desorbed from the weak and moderate/strong basic sites, respectively (Table S1), which was found to be optimized for the 20%Ga_2_O_3_-Al_2_O_3_ catalyst. Although the CO_2_ adsorption was expected to decrease as the specific surface area decreases, the observed trend of CO_2_ adsorption presented in Table S1 should not only be related to the variation in the specific surface area but also to the interactions between the Ga_2_O_3_ and the Al_2_O_3_ support induced by the increase in Ga_2_O_3_ content. Since the specific surface area of the investigated catalysts was significantly varied from 4 to 64.4 m^2^ g^−1^, the results of Table S1 were normalized by the specific surface area in order not to contain contributions from the variation in this parameter. It was found that the amount of CO_2_ desorbed (in μmol m^−2^) from both the weak and moderate/strong basic sites was maximized for the sample containing 20 wt.% Ga_2_O_3_ (Table 2). This can be clearly seen in Figure 3, where the total amount of CO_2_ desorbed during TPD was plotted as a function of the Ga_2_O_3_ content. Specifically, the amount of CO_2_ was found to increase from 0.54 m^2^ g^−1^ for the bare Al_2_O_3_ to 1.54 m^2^ g^−1^ for the 20%Ga_2_O_3_-Al_2_O_3_ catalyst and subsequently decreased to 1.08 m^2^ g^−1^ with the progressive increase in Ga_2_O_3_ content to 40 wt.%, while it was further decreased to 0.75 m^2^ g^−1^ for the bare Ga_2_O_3_.

The results of Figure 3 provide evidence that the surface basicity of Ga_2_O_3_-Al_2_O_3_ catalysts depends strongly on the Ga_2_O_3_ concentration, which is in accordance with previous studies. For example, Li et al. [33], who investigated the surface basicity of x%Ga_2_O_3_-ZrO_2_ (x: 0, 5, 10, 15, 20 wt.%) catalysts by CO_2_-TPD, found that a maximum number of basic sites appeared for Ga_2_O_3_ content of 15 wt.%. Moreover, Michorczyk et al. [34] reported that the density of basic sites on the surface of Ga_2_O_3_-Al_2_O_3_ catalysts increased with increasing Ga_2_O_3_ loading from 0 to 20 wt.%, in excellent agreement with the results of the present study. The adsorption of CO_2_ was also found to be facilitated by increasing the concentration of Ga_2_O_3_ over Ni/Ga_2_O_3_-Al_2_O_3_ catalysts [31]. Similarly, Orlyk et al. [32] demonstrated that the total surface basicity of GaxSiBEA (x: 1, 2, 4 wt.%) zeolites increased almost proportionally to the content of Ga. Furthermore, the addition of Ga_2_O_3_ on Ce_0_._6_Zr_0_._4_O_2_ with loadings varying between 0 and 15 wt.% was found to enhance the surface basicity, which was optimized over the sample containing 5 wt.% Ga_2_O_3_ [35].

The CO_2_ adsorption/desorption characteristics were also investigated by in situ FTIR spectroscopy, and the results obtained are presented in Figure S3. The DRIFT spectrum collected at 25 °C in He flow for bare Al_2_O_3_ (Figure S3a) following its interaction with 5%CO_2_ (in He) was consisted of various bands in the 1700–1200 cm^−1^ region previously attributed to bicarbonate species (1658, 1433 and 1229 cm^−1^), as well as to unidentate and bidentate carbonates (1627, 1558 and 1373 cm^−1^) [36,37,38,39,40,41,42]. According to previous studies, the formation of carbonate-like species on the catalyst surface occurs via CO_2_ interaction with the basic sites of the metal oxide, i.e., the surface hydroxyl groups and/or the low-coordination oxygen anions [43,44]. In particular, it was suggested that CO_2_ interaction with the surface OH groups is responsible for bicarbonate formation, while unidentate, bidentate, and bridged carbonate species are mainly generated by CO_2_ interaction with oxygen anions [36,39,40,43,45,46]. Increase in temperature under He flow led to a decrease in the intensity of all bands, which almost disappeared above 250 °C, implying that the corresponding species were desorbed from the alumina surface (Figure S3a).

Similar bands were detected in the spectra obtained from the Ga_2_O_3_-modified Al_2_O_3_ catalysts following CO_2_ adsorption, implying that the same surface species were formed independently of the Ga_2_O_3_ content (Figure S3b–e). It should be noted that CO_2_ adsorption on Ga_2_O_3_ surface was previously found to result in the formation of bicarbonates and bidentate carbonates, giving rise to the development of bands located at wavenumbers close to those discussed above for Al_2_O_3_ [41,42,47]. Therefore, part of the detected bands in the FTIR spectra may be related to carbonate-like species associated with Ga_2_O_3_ particles. The shoulder appeared at 1690 cm^−1^ for the sample containing 10 wt.% Ga_2_O_3_ was previously attributed to bidentate carbonates adsorbed on Al_2_O_3_ surface or bridged carbonates adsorbed on Ga_2_O_3_ surface [42,47]. This band may also be present in the spectra obtained from the rest of the catalysts examined but cannot be distinguished due to the coexistence of more than one band in the corresponding wavenumber region. It is of interest to note that the relative population of surface species seems to be maximized for the 20%Ga_2_O_3_-Al_2_O_3_ catalyst and eliminated above 250 °C for all composite metal oxides (Figure S3), in excellent agreement with the results of CO_2_-TPD experiments discussed above (Figure 2, Table 2).

Concerning the spectra obtained from the bare Ga_2_O_3_, only two weak peaks were detected at 1621 and 1333 cm^−1^ due to bicarbonate and bidentate carbonate species, respectively, which desorbed from the catalyst surface below 200 °C [41,42,47]. The low CO_2_ adsorption capacity of this sample may be correlated with its low specific surface area and agrees well with the results of Figure 2.

The surface acidity of the investigated metal oxides was examined by the potentiometric titration method described above, and the results obtained are presented in Figure S4, where the potentiometric titration curves of the dried catalysts and the control sample, fitted by the Boltzmann function, are presented along with the corresponding Gran’s function plots and their linearization. Based on the Gran’s method described above, two equivalence points were determined for each catalyst, *V_a_* and *V_b_*, which represent the equivalence volumes obtained from the acidic and basic slopes of the Gran’s function, respectively. Two similar equivalence points were also extrapolated, *V_aN_* and *V_bN_*, in the case of the control sample (0.05 M KNO_3_).

The concentration of the different types of acid sites was estimated by the intersections of the straight lines with the Volume axis using the Equations (6)–(8) and results obtained (Table S2, in μmol·g^−1^) were normalized with respect to the specific surface area of each catalyst and presented in Table 3 and Figure S5 (in μmol·m^−2^). It was found that bare Al_2_O_3_ and x%Ga_2_O_3_-Al_2_O_3_ catalysts consisted of three different types of acid sites: very weak, weak, and strong acid sites. The density of very weak (A_vw_) and strong acid sites (A_s_) was, generally, maximized for the sample containing 30 wt.% Ga_2_O_3_, while that of weak acid sites (A_w_) was progressively increased with increasing the Ga_2_O_3_ content from 10 to 40 wt.% and found to be lower than that of bare Al_2_O_3_ in the case of the 10, 20 and 30 wt.% Ga_2_O_3_-Al_2_O_3_. Only weak and strong acid sites were determined over bare Ga_2_O_3_, which, given its low specific surface area, exhibited significantly higher A_w_ and A_s_ values (in μmol·m^−2^) than the rest of the catalysts examined. The total surface acidity of the investigated catalysts, defined as the sum of A_vw_, A_w_, and A_s_ values, was found to gradually increase from 6.64 to 85.00 μmol·m^−2^ with increasing Ga_2_O_3_ content from 0 to 100 wt.% (Table 3, Figure 3).

**Figure 4:**
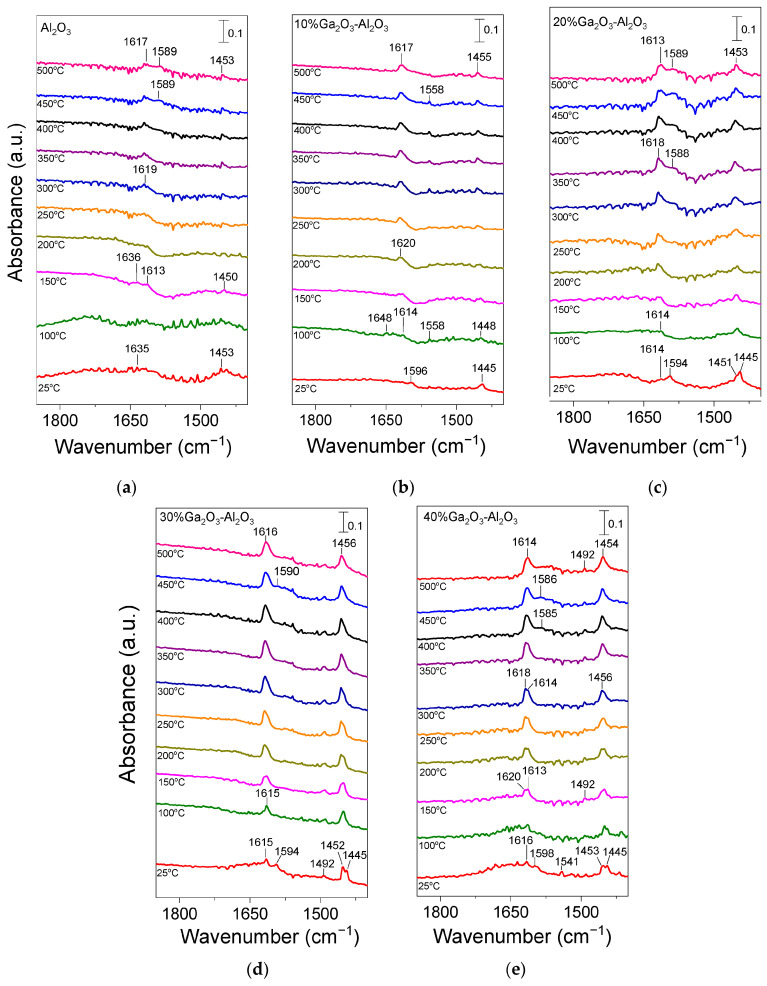
DRIFT spectra obtained from (**a**) Al_2_O_3_, (**b**) 10%Ga_2_O_3_-Al_2_O_3_, (**c**) 20%Ga_2_O_3_-Al_2_O_3_, (**d**) 30%Ga_2_O_3_-Al_2_O_3_ and (**e**) 40%Ga_2_O_3_-Al_2_O_3_ catalysts after pyridine adsorption at 25 °C for 2 h and subsequent stepwise heating up to 500 °C under He flow.

In order to obtain additional insight related to the type and strength of acid sites on the surface of the investigated catalysts, pyridine adsorption/desorption experiments using FTIR spectroscopy were applied. Results obtained are presented in Figure 4. Pyridine adsorption on bare Al_2_O_3_ (Figure 4a) resulted in the development of two bands at 1635 and 1453 cm^−1^ in the spectrum recorded at 25 °C, which can be attributed to pyridine species interacting with Brønsted and strong Lewis acid sites, respectively [3,27,28,48,49,50,51,52,53,54,55]. The former band disappeared at temperatures higher than 150 °C, while the latter one was present in all spectra collected up to 500 °C, indicating that the corresponding species were adsorbed strongly on the alumina surface. It should be noted, however, that the band at 1453 cm^−1^ may also contain contributions from physisorbed pyridine at least at low desorption temperatures [48,51,56]. A new band was discerned at ca. 1613 cm^−1^ in the spectrum obtained at 150 °C, which was accompanied by the parallel development of a broad shoulder at ca. 1589 cm^−1^ at temperatures higher than 400 °C. Both bands were detectable up to 500 °C and can be assigned to pyridine adsorption on strong (1613 cm^−1^) and weak/moderate (1589 cm^−1^) Lewis acid sites [28,48,50,52,53,55,56].

No significant variations were observed in the spectra obtained from the 10%Ga_2_O_3_-Al_2_O_3_ catalyst (Figure 4b), besides (a) the appearance of a band at 1596 cm^−1^ in the spectrum collected at 25 °C, which was diminished at higher temperatures and was previously attributed to physiosorbed or H-bonded pyridine [48,51,56], (b) the detection of a weak band at 1558 cm^−1^ at 100 °C, which was present in all spectra collected up to 450 °C and was due to pyridine protonated by strong Brønsted acid sites [28,48,50] and (c) the absence of the band discussed above at 1589 cm^−1^ corresponding to weak/moderate Lewis acid sites. The bands assigned to pyridine species adsorbed on Lewis acid sites exhibited significantly higher intensity than those assigned to pyridine species adsorbed on Brønsted acid sites and were present up to 500 °C, implying that both the number and strength of Lewis acid sites were higher. Further increase in Ga_2_O_3_ content up to 40 wt.% led to a progressive increase in features owing to pyridine adsorbed on Lewis acid sites (1613–1615, 1585–1590 and 1451–1453 cm^−1^) while no characteristic peaks associated with Brønsted acidity (1640 or 1540–1560 cm^−1^) was discerned in any of the samples containing 20, 30 and 40 wt.% Ga_2_O_3_ (Figure 4c–e). A new band can be discerned at 1492 cm^−1^ in the spectra obtained from the 30%Ga_2_O_3_-Al_2_O_3_ (Figure 4d) and 40%Ga_2_O_3_-Al_2_O_3_ (Figure 4e) catalysts, which was previously reported to contain overlapping bands due to pyridine adsorption on both Lewis and Brønsted acid sites [48,55]. Results of Figure 4 indicate that even if the Ga_2_O_3_-Al_2_O_3_ catalysts contained Brønsted acid sites, both their number and strength were notably lower compared to those of Lewis acid sites and were eliminated with increasing gallium oxide loading. It is generally accepted that Lewis acid sites in Ga_2_O_3_-based catalysts are related to coordinatively unsaturated Ga^3+^ ions in the tetrahedral position, while Brønsted acid sites are related to Ga-OH groups on the catalyst surface [28,55,56,57]. It has also been proposed that the Lewis acid sites over Ga_2_O_3_-SiO_2_ catalysts originate from Ga_2_O_3_ particles that have not been incorporated into the SiO_2_ framework, while Ga_2_O_3_ particles incorporated into the SiO_2_ framework are responsible for the creation of Brønsted acid sites [57]. Therefore, taking into account that the population of Lewis acid sites was higher than Brønsted acid sites for the Ga_2_O_3_-Al_2_O_3_ catalysts investigated in the present study, it can be assumed that gallium oxide particles mainly remained on the surface of alumina rather than incorporated into its framework. Although part of Ga_2_O_3_ may be incorporated into the Al_2_O_3_ structure, as evidenced by the small shift of the diffraction peak located at 2θ = 67.4° observed in X-ray diffractograms (Figure 1B), the fraction of Ga_2_O_3_ particles that remained on the catalyst surface seems to be higher.

It should also be noted that, in the case of catalysts containing 20, 30, and 40 wt.% Ga_2_O_3_, two overlapping features can be discerned in the 1445–1453 cm^−1^ region. The one located at ca. 1452 cm^−1^ was associated with strong Lewis acid sites, while that detected at ca. 1445 cm^−1^ was associated with physisorbed pyridine, which in all cases disappeared upon heating the catalyst at 100 °C. This was also the case for the 1594–1598 cm^−1^ band, which, for all gallium oxide containing catalysts, was only present at 25 °C, further supporting the above suggestion that it was related to physisorbed pyridine. Concerning the bare Ga_2_O_3_ sample, although a similar pyridine adsorption/desorption experiment was conducted, no clear peaks could be discerned, most likely due to the significantly lower specific surface area (4 m^2^ g^−1^) of this sample.

Results of Figure 4 clearly indicate that the population of pyridine adsorbed surface species, and therefore, the number of acid sites on the catalyst surface, increased significantly with increasing Ga_2_O_3_ content from 0 to 40 wt.% and are in excellent agreement with the results of Table 3 obtained from the potentiometric titration experiments. The increase in the acid site density with the addition of Ga_2_O_3_ on Al_2_O_3_, TiO_2_, and SiO_2_ supports was also reported in our previous studies [4,5,8]. Moreover, Zhou et al. [50] demonstrated that the total surface acidity increased with the addition of Ga_2_O_3_ on Al_2_O_3_, with the distribution of acid sites, however, remaining unchanged, whereas Chen et al. [28] found a greater Lewis acid site density over spinel-type gallia–alumina solid solution Ga*_x_*Al_10−_*_x_*O_15_ (*x*: 0–10) oxides compared to bare alumina. In addition, Ga_2_O_3_-Al_2_O_3_, prepared by the atomic layer deposition method, was found to be characterized by higher gallium oxide dispersion and stronger interaction with the alumina support, which was able to form more Ga-O-Al linkages and lead to higher surface acidity [2]. An increase in the number of weak acid sites was also observed by Ga_2_O_3_ deposition on the SiO_2_ surface [19]. Moreover, Castro-Fernandez et al. [58] studied the coordination geometry and Lewis acidity of surface sites over gallia–alumina oxides and proposed that the optimization of the Ga/Al atomic ratio is able to adjust the relative abundance and strength of Ga-related Lewis surface acid sites. They found that Ga-rich samples exhibited a higher fraction of six-coordinated Ga sites, as well as a higher Ga related strong Lewis acidity, in agreement with the results of the present study.

In order to investigate the reducibility of supported Ga_2_O_3_ catalysts, H_2_-TPR experiments were conducted over the 10%Ga_2_O_3_-Al_2_O_3_ and 30% Ga_2_O_3_-Al_2_O_3_ samples (Figure S6). No reduction peaks were observed in the H_2_-TPR profile of the 10%Ga_2_O_3_-Al_2_O_3_ catalyst, indicating that this catalyst was not able to be reduced by hydrogen. Increase in Ga_2_O_3_ content to 30 wt.% led to the appearance of a single weak peak in the H_2_-TPR profile centered at ~180 °C, which can be attributed to the reduction of well-dispersed Ga particles and/or GaO^+^ species interacting with the support [17,59]. The total amount of hydrogen consumed during the H_2_-TPR experiment was estimated by integrating the area below the H_2_ response curve and found to be 27.9 μmol g^−1^. Contradicting results have been reported in the literature related to the reducibility of supported Ga_2_O_3_ catalysts. For example, treatment of Ga_2_O_3_-SiO_2_ catalyst with H_2_ led to the appearance of peaks at low binding energies in the XPS spectra previously ascribed to the formation of gallium hydrides, Ga^2+^ or Ga^+^ species, implying that the reduction of Ga^3+^ is feasible [16]. This was also the case for Ga_2_O_3_-Al_2_O_3_ and Ga_2_O_3_-ZSM-5 catalysts explored by H_2_-TPR experiments [17]. The reduction ability of gallium from Ga^3+^ to Ga^+^ in H_2_ atmosphere were also studied employing X-ray absorption near edge spectroscopy (XANES) and corroborated by an observed shift of the XANES edge energy upon exposure of Ga-H-ZSM5, Ga–H-BEA and Ga–H-ZSM5 to H_2_ at 500–550 °C [60,61,62,63]. Contrarily, Getsoian et al. [13] demonstrated that Ga^3+^ is not reduced to Ga^+^ when Ga–SiO_2_ and Ga–H-BEA catalysts are exposed to hydrogen at high temperature. Results presented in Figure S6 clearly indicate that the reducibility of Ga_2_O_3_-Al_2_O_3_ catalysts is generally limited, but it can be slightly enhanced with increasing Ga_2_O_3_ content.

### 3.2. Catalytic Performance Tests for the CO_2_-ODP Reaction

**Figure 5:**
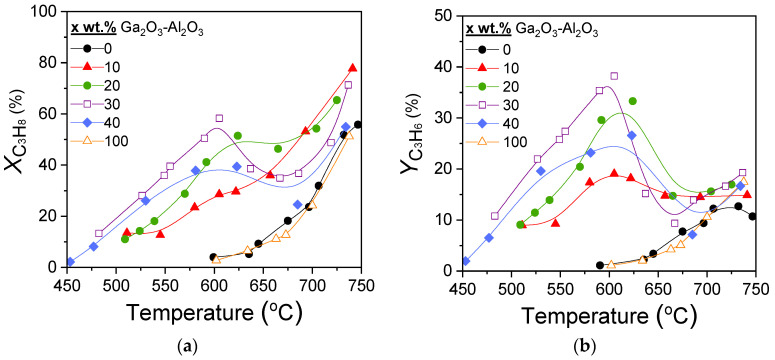
Effect of reaction temperature on the (**a**) conversion of propane and (**b**) propylene yield obtained over Al_2_O_3_, Ga_2_O_3_, and x%Ga_2_O_3_-Al_2_O_3_ catalysts. Experimental conditions: Mass of catalyst: 500 mg; particle diameter: 0.15 < d_p_ < 0.25 mm; Feed composition: 5% C_3_H_8_, 25% CO_2_ (balance He); Total flow rate: 50 cm^3^ min^−1^.

Results of catalytic performance experiments carried out over the x%Ga_2_O_3_-Al_2_O_3_ catalysts for the CO_2_-ODP reaction are presented in Figure 5. It was observed that both propane conversion (Figure 5a) and propylene yield (Figure 5b) of the composite metal oxides were, for all Ga_2_O_3_ loadings, higher than that of bare Al_2_O_3_ and Ga_2_O_3_. At temperatures lower than 600 °C, $X_{C_{3}H_{8}}$ and $Y_{C_{3}H_{6}}$ increased with increasing Ga_2_O_3_ content from 0 to 30 wt.%, while they were decreased with the addition of 40 wt.%Ga_2_O_3_, as well as for the bare Ga_2_O_3_, which exhibited identical performance with that of bare Al_2_O_3_. The most active 30%Ga_2_O_3_-Al_2_O_3_ catalyst was activated above 475 °C and reached maximum $X_{C_{3}H_{8}}$ = 58% and $Y_{C_{3}H_{6}}$ = 39% at 605 °C. It is of interest to note that the samples containing 20, 30, and 40 wt.% Ga_2_O_3_ presented a decrease in both $X_{C_{3}H_{8}}$ and $Y_{C_{3}H_{6}}$ in the temperature range of ~600–670 °C, which was then increased again with further increase in temperature to 750 °C. This behavior—which was not observed for the 10 wt.% Ga_2_O_3_-Al_2_O_3_, Al_2_O_3 _and Ga_2_O_3_ samples—was more intense as Ga_2_O_3_ loading was becoming higher and, as it will be discussed below, can be attributed to side reactions occurring in parallel, hindering propylene generation.

**Figure 6:**
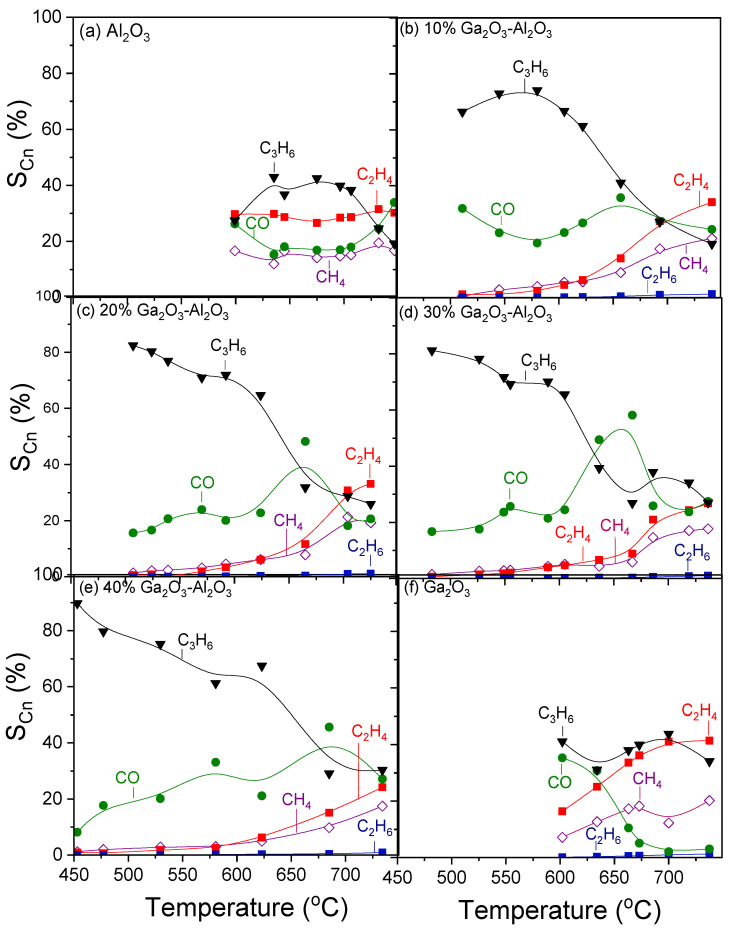
Selectivities towards reaction products as a function of reaction temperature obtained over the (**a**) Al_2_O_3_, (**b**) 10%Ga_2_O_3_-Al_2_O_3_, (**c**) 20%Ga_2_O_3_-Al_2_O_3_, (**d**) 30%Ga_2_O_3_-Al_2_O_3_, (**e**) 40%Ga_2_O_3_-Al_2_O_3_, and (**f**) Ga_2_O_3_ catalysts. Experimental conditions: same as in Figure 5.

Figure 6 shows the selectivities towards reaction products as a function of temperature for the investigated catalysts. In the case of bare alumina (Figure 6a), propylene selectivity ($S_{C_{3}H_{6}}$) increased from 27 to 43% with increasing temperature from 600 to 635 °C, respectively, remained almost constant with further increase in temperature to 700 °C, whereas it was subsequently decreased to 19% with gradual increase in temperature to 750 °C. Selectivity towards CO ($S_{CO}$) followed the opposite trend, taking, generally, lower values varying between 17 and 34%. In addition to C_3_H_6_ and CO, C_2_H_4_ and CH_4_ were also detected with their selectivities ($S_{C_{2}H_{4}}$ and $S_{CH_{4}}$) remaining almost stable in the entire temperature range examined at 27–30% and 14–18%, respectively. The addition of Ga_2_O_3_ (Figure 6b–e) resulted in a significant increase in $S_{C_{3}H_{6}}$, which reached 90% at ~450 °C for the sample containing 40 wt.% (Figure 6e). An increase in the reaction temperature led to a progressive decrease in $S_{C_{3}H_{6}}$, which became more intense in the temperature range of 600–670 °C, where, as mentioned above, $X_{C_{3}H_{8}}$ and $Y_{C_{3}H_{6}}$ presented a sharp decrease over the 20, 30, and 40%Ga_2_O_3_-Al_2_O_3_ catalysts. In the same temperature range, $S_{CO}$, which showed, in general, a mild upward trend with temperature, exhibited an abrupt increase, which was always followed by a decrease at the same levels as those obtained below 600 °C. Interestingly, C_2_H_4_ and CH_4_ formation were limited below 600 °C for all composite metal oxides, while their production was enhanced at higher temperatures, with the corresponding selectivities, at a given temperature, decreasing as Ga_2_O_3_ content was increased from 10 to 40 wt.%. In contrast, bare Ga_2_O_3_ exhibited the highest values of $S_{C_{2}H_{4}}$ and $S_{CH_{4}}$ in the entire temperature range examined, and the lowest $S_{CO}$ at elevated temperatures (Figure 6f).

The addition of Ga_2_O_3_ also led to the formation of traces of C_2_H_6_ due to propane hydrogenolysis:

(12) $$C_{3}H_{8}+\;H_{2}\;↔\;C_{2}H_{6}+\;{\mathrm{CH}}_{4}\;{\Delta H}_{298Κ}^{0}=-55.4\;\mathrm{kJ}/\mathrm{mol}$$

The decrease in $X_{C_{3}H_{8}}$ and $Y_{C_{3}H_{6}}$ between 600 and 670 °C can be attributed to the reaction of propylene decomposition, which may be partially responsible for the observed production of CH_4_ and C_2_H_4_:

(13) $$2C_{3}H_{6}\;↔\;2{\mathrm{CH}}_{4}+C_{2}H_{4}+2C_{\left(s\right)}\;{\Delta H}_{298Κ}^{0}=-137.6\mathrm{kJ}/\mathrm{mol}$$

Taking into account that $S_{C_{2}H_{4}}$ was always higher than that of $S_{CH_{4}}$, part of these compounds may also be produced via the following reaction:

(14) $$2{\mathrm{CO}}_{2}+2C_{3}H_{8}\;↔\;3C_{2}H_{4}+2\mathrm{CO}\;+2H_{2}O\;{\Delta H}_{298Κ}^{0}=447.2\;\mathrm{kJ}/\mathrm{mol}$$

whereas the reactions of propane decomposition (15) and (16) and/or propane hydrogenolysis (17) cannot be excluded:

(15) $$C_{3}H_{8}\;↔\;C_{2}H_{4}+\;\mathrm{CH}4\;{\Delta H}_{298Κ}^{0}=81.7\;\mathrm{kJ}/\mathrm{mol}$$

(16) $$C_{3}H_{8}\;↔\;{\mathrm{CH}}_{4}+2H_{2}+2C_{\left(s\right)}\;{\Delta H}_{298Κ}^{0}=29.2\;\mathrm{kJ}/\mathrm{mol}$$

(17) $$C_{3}H_{8}+2H_{2}↔\;3{\mathrm{CH}}_{4}\;{\Delta H}_{298Κ}^{0}=-120.0\;\mathrm{kJ}/\mathrm{mol}$$

Reaction (14) in combination with the reverse WGS (2) and reverse Boudouard (3) reactions may also contribute to the notable increase in $S_{CO}$ between 600 and 670 °C. High reaction temperatures are also known to favor the reaction of dry propane reforming (18), which also favors the formation of CO:

(18) $$C_{3}H_{8}+3{\mathrm{CO}}_{2}↔6\mathrm{CO}\;+4H_{2}\;{\Delta H}_{298Κ}^{0}=644.1\;\mathrm{kJ}/\mathrm{mol}$$

**Figure 7:**
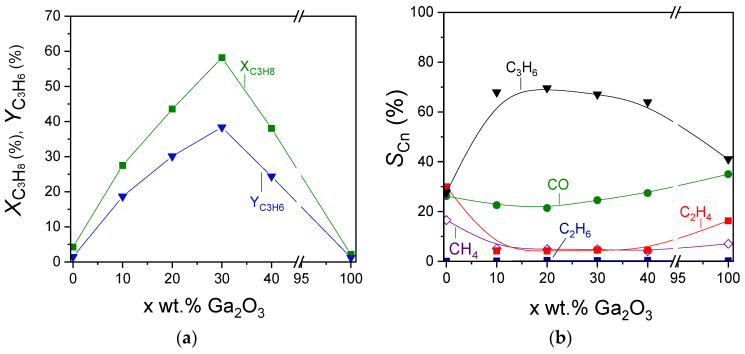
Effect of Ga_2_O_3_ content on the (**a**) propane conversion and propylene yield, and (**b**) selectivities towards reaction products measured at 600 °C for the CO_2_-ODP reaction.

The effect of Ga_2_O_3_ content on the propane conversion, propylene yield, and product selectivities can be better seen in Figure 7, where the corresponding measurements were obtained at 600 °C. Catalytic activity was optimized in the presence of 30 wt.% Ga_2_O_3_. Specifically, $X_{C_{3}H_{8}}$ and $Y_{C_{3}H_{6}}$ were remarkably increased from 4 to 58% and from 1.5 to 39%, respectively, with increasing Ga_2_O_3_ content from 0 to 30 wt.%, followed by their gradual decrease to the initial values with further increase in Ga_2_O_3_ content to 40 and 100 wt.% (Figure 7a). Interestingly, $S_{C_{3}H_{6}}$ at 600 °C increased significantly from 28% for bare Al_2_O_3_ to ~68% for the samples containing 10%, 20%, and 30%Ga_2_O_3_ and decreased to 64 and 41% for the 40%Ga_2_O_3_-Al_2_O_3_ and bare Ga_2_O_3_, respectively (Figure 7b). The opposite trend was observed for $S_{C_{2}H_{4}}$ and $S_{CH_{4}}$ measured at 600 °C, which were minimized to the same value of ~4.5% for all composite metal oxides, while higher values were obtained for the bare metal oxides ($S_{C_{2}H_{4}}$ = 17% and $S_{CH_{4}}$ = 30% for Al_2_O_3_, $S_{C_{2}H_{4}}$ = 16.5% and $S_{CH_{4}}$ = 7% for Ga_2_O_3_). The effect of Ga_2_O_3_ on $S_{CO}$ was less important (ranging between 21 and 35%), most possibly because, as discussed above, CO may originate from various reactions under CO_2_-ODP conditions (reactions (1)–(3), (14) and (18)), which may be affected in a different manner by Ga_2_O_3_ loading. Therefore, the increase in $S_{CO}$ induced by some of these reactions may be balanced by the decrease in $S_{CO}$ caused by others, thus leading to relatively low fluctuations.

Results of Figure 5, Figure 6 and Figure 7 clearly indicate that the catalytic activity is strongly affected by the concentration of Ga_2_O_3_, which is not only able to increase propane conversion to propylene but also to suppress side product formation at temperatures of practical interest (<600 °C). This can also be seen in Figure S7a, where the ratio of $\;S_{C_{3}H_{6}}$/ $S_{C_{2}H_{4}}$ at 600 °C was plotted as a function of Ga_2_O_3_ content. As it is observed, the $S_{C_{3}H_{6}}$/ $S_{C_{2}H_{4}}$ ratio goes through a maximum value of 17 for the 20%Ga_2_O_3_-Al_2_O_3_ catalyst, which was 18- and ~7-fold higher than those measured for the corresponding bare Al_2_O_3_ and Ga_2_O_3_, respectively. This implies that the C–H bond cleavage against that of the C–C bond can be optimized with the addition of a suitable amount of Ga_2_O_3_ content.

**Figure 8:**
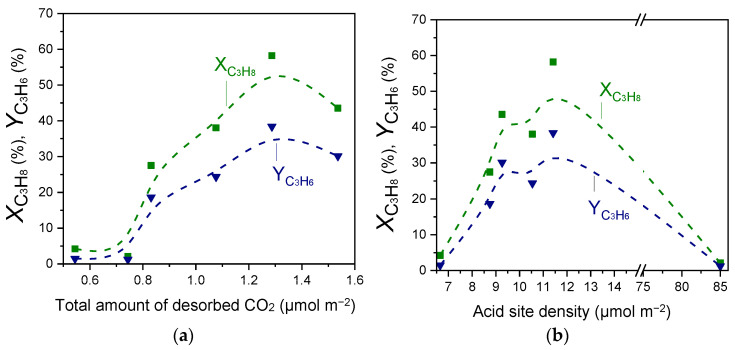
Propane conversion and propylene yield measured at 600 °C for the CO_2_-ODP reaction as a function of the (**a**) total amount of desorbed CO_2_ during CO_2_-TPD experiments and (**b**) the acid site density obtained over Al_2_O_3_, Ga_2_O_3,_ and x%Ga_2_O_3_-Al_2_O_3_ catalysts.

Results of Figure 2, Figure 3 and Figure 4 and Table 2 and Table 3 showed that the acid site density was progressively increased with increasing Ga_2_O_3_ content, while a volcano-type behavior was found to exist between this parameter and surface basicity, with the maximum value being observed for the 20%Ga_2_O_3_-Al_2_O_3_ catalyst. In an attempt to understand the effect of acid/base properties on catalytic activity, $X_{C_{3}H_{8}}$ and $Y_{C_{3}H_{6}}$ measured at 600 °C were plotted as a function of the total amount of CO_2_ desorbed during CO_2_-TPD (Table 2) and the acid site density measured by the potentiometric titration measurements (Table 3). Results are presented in Figure 8, where a noteworthy correlation was found to exist. Specifically, $X_{C_{3}H_{8}}$ and $Y_{C_{3}H_{6}}$ exhibited optimum values for intermediate values of surface basicity and acidity, which both correspond to the sample containing 30 wt.% Ga_2_O_3_. This is in agreement with our previous studies over M_x_O_y_-TiO_2_ (M: Zr, Ce, Ca, Cr, Ga) and M_x_O_y_-SiO_2_ (M: Ca, Sn, Cr, Ga) catalysts [4,5], providing evidence that the number of both acid and basic sites determines the CO_2_-ODP activity. Comparing the results of Figure 8 with those presented in Figure 2 and Figure S5 shows that catalytic activity was mainly determined by the strong acidic and the weak basic sites of the catalyst surface.

The surface basicity was also found to influence the $S_{C_{3}H_{6}}/S_{C_{2}H_{4}}$ ratio, which was significantly higher for all Ga_2_O_3_-Al_2_O_3_ catalysts and optimum for the 10% and 20%Ga_2_O_3_-Al_2_O_3_ catalysts, ($S_{C_{3}H_{6}}$/$S_{C_{2}H_{4}}≅17$), compared to the bare single metal oxides characterized by lower surface basicity (Figure S7b). On the other hand, the $S_{C_{3}H_{6}}/S_{C_{2}H_{4}}$ ratio exhibited a volcano-type correlation with respect to the acid site density, with the 10% and 20%Ga_2_O_3_-Al_2_O_3_ catalysts presenting the maximum values ($S_{C_{3}H_{6}}/S_{C_{2}H_{4}}≅17$) (Figure S7c). Although the aforementioned optimum $S_{C_{3}H_{6}}$/$S_{C_{2}H_{4}}$ ratios were not observed for the most active 30%Ga_2_O_3_-Al_2_O_3_ catalyst, results clearly indicate that the C–H bond cleavage was facilitated compared to C–C bond break over samples characterized by moderate surface basicity and acidity. Based on the above, the acid/base properties of catalysts can be considered as the key physicochemical properties for the CO_2_-ODP reaction.

An optimum propane dehydrogenation activity for intermediate Ga-related Lewis surface acidity, which was achieved by optimizing the Ga/Al atomic ratio, was also reported by Castro-Fernandez et al. [58]. Among various Ga/Al atomic ratios examined (1:6, 1:3, 3:1, and 1:0), superior catalytic activity, propylene selectivity, and stability were obtained for Ga/Al = 1:3, which were attributed to the higher abundance of four-coordinated Ga sites and the higher relative number of weak/medium Lewis acid sites. The increase in Ga_2_O_3_ loading from 1 to 9 wt.% was found to increase the fraction of gallium in the oxidized state over xGa_2_O_3_/SBA-15 catalysts, with the TOF of propane conversion, however, being maximized for an intermediate Ga_2_O_3_ loading (5 wt.%) [17]. An intermediate Ga/Al ratio equal to 3:8 of Ga_2_O_3_-Al_2_O_3_ nanofibers was also reported to present superior $X_{C_{3}H_{8}}$ and $S_{C_{3}H_{6}}$ of 48.4 and 96.8%, respectively, for the CO_2_-assisted oxidative dehydrogenation of propane at 500 °C [3]. Moreover, Tedeeva et al. [19] pointed out the importance of the acid sites in achieving high catalytic activity for the propane dehydrogenation in the presence of CO_2_ over Ga/SiO_2_ catalysts. The authors studied catalysts with different Ga content in the range of 3–50 wt.% dispersed on three different SiO_2_ powders characterized by different textural properties, and they found that both the nature and texture of the support, as well as the Ga content, influence catalytic activity. The best results ($X_{C_{3}H_{8}}$ = 33% and $S_{C_{3}H_{6}}$ = 84% at 650 °C) were obtained when Ga_2_O_3_ oxide with Ga content of 7 wt.% was supported on the SiO_2_ powder characterized by the highest specific surface area, which also exhibited a higher number of Brønsted acid sites. Besides the high initial activity of this sample, a decrease in $X_{C_{3}H_{8}}$ was observed after 10 h on stream which, was stabilized at 20% from 10 to 20 h of continuous operation. The higher surface total moderate acidity of alumina supported Ga_2_O_3_ catalysts synthesized by the atomic layer deposition method was also found to facilitate the conversion of C_3_H_8_ to C_3_H_6_ by decreasing the energy barrier for the activation of C-H bond [2]. The effect of Ga loading was investigated in the range of 1–2.9 wt.%, and results showed that the sample containing 2.9 wt.% Ga presented the highest performance, which, although it was drastically deactivated during the first 45 min on stream, $X_{C_{3}H_{8}}$ and $S_{C_{3}H_{6}}$ were stabilized at 38 and 82%, respectively, at 600 °C for the next ~2 h. It is of interest to note that propane conversion and propylene yield obtained in the present study for the 30%Ga_2_O_3_-Al_2_O_3_ catalyst ($X_{C_{3}H_{8}}$ = 59% and $Y_{C_{3}H_{6}}$ = 39% at ~600 °C) was higher than most of those reported thus far in the literature, enabling the operation of the reaction at low temperatures which besides the advantage of inhibiting side reactions, offer the benefit of low energy requirements and thus, low operational cost.

### 3.3. TOS Stability Tests over 30%Ga_2_O_3_-Al_2_O_3_

**Figure 9:**
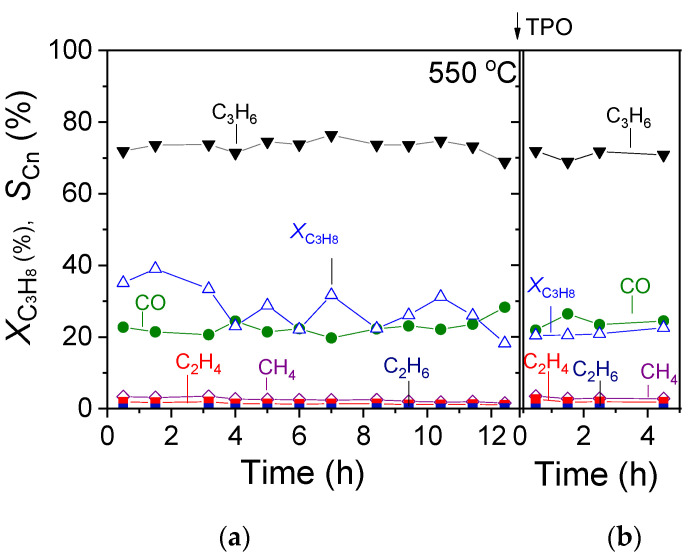
TOS stability test conducted at 550 °C under CO_2_-ODP conditions over the (**a**) fresh and (**b**) spent 30%Ga_2_O_3_-Al_2_O_3_ catalyst following the TPO experiment.

**Figure 10:**
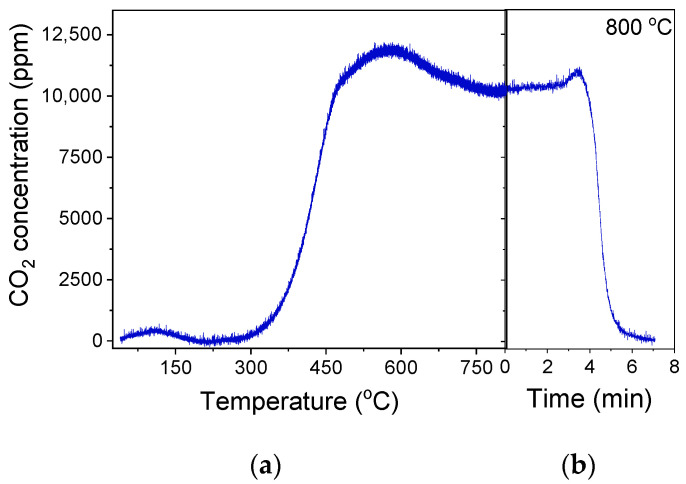
(**a**) Responses of CO_2_ produced during the TPO experiment occurred after the TOS stability tests conducted at 550 °C, as presented in Figure 9a over the 30%Ga_2_O_3_-Al_2_O_3_ catalyst. In (**b**), the CO_2_ response at 800 °C was recorded as a function of time until complete oxidation of carbon.

The most effective 30%Ga_2_O_3_-Al_2_O_3_ catalyst was subjected to a time on stream stability test for a period of ~12 h at a constant temperature of 550 °C, and results are presented in Figure 9a in terms of C_3_H_8_ conversion ($X_{C_{3}H_{8}}$) and product selectivity ($S_{C_{n}}$) versus time. It was observed that after an initial period of 4 h on stream, where $X_{C_{3}H_{8}}$ progressively decreased from 35 to 23%, it exhibited a rather stable performance, periodically fluctuating between 22 and 32% until the end of the stability test. Interestingly, the selectivity towards reaction products remained constant during the entire time of the experiment, taking values of $S_{C_{3}H_{6}}$ = 72%, $S_{CO}$ = 22%, $S_{CH_{4}}$ = 2.5%, $S_{C_{2}H_{4}}$ = 1.2% and $S_{C_{2}H_{6}}$ < 1%. In order to investigate the potential carbon deposition on the catalyst surface during this experiment, the temperature was decreased to 25 °C in He flow, and the catalyst was exposed to 1%O_2_ (in He) flow, followed by a TPO experiment using a heating rate of 10 °C/min. Results (Figure 10) showed that CO_2_ started to elute above 40 °C, giving rise to a weak peak centered at 110 °C, followed by a major peak above 300 °C with a maximum at ~580 °C. The oxidation of carbon was not completed when the temperature reached 800 °C, and thus, the catalyst remained at this temperature for ~6 min until the CO_2_ response returned to the baseline. The amount of coke formed was estimated by integrating the area below the CO_2_ response curve versus time and found to be 2950.8 μmol·g^−1^. Results indicate that despite the significant amount of the so-formed carbon, the 30%Ga_2_O_3_-Al_2_O_3_ catalyst exhibited a sufficiently stable performance for 12 h on stream. When the TPO experiment was completed, the catalyst was again exposed to the reaction mixture at 550 °C for 5 h, and the results (Figure 9b) showed that the values of both $X_{C_{3}H_{8}}$ and $S_{C_{n}}$ were identical to those presented in Figure 9a.

Similar experiments were carried out at 600 and 650 °C, aiming to explore the effect of reaction temperature on the catalyst’s stability and tendency towards carbon formation. It was found that the catalyst interaction with the gas stream at 600 °C led to a gradual decrease in the propane conversion from 56 to 13% within the first 7.5 h on stream, which then remained almost constant up to 12.5 h (Figure S8a). This decrease was accompanied by a decrease in $S_{C_{3}H_{6}}$ (from 61 to 37%) and a parallel increase in $S_{CO}\;$(from 28 to ~38%), $S_{CH_{4}}$ (from 4 to 8.3%) and $S_{C_{2}H_{4}}$ (from 4.9 to 15%), indicating that the oxidative dehydrogenation of propane was hindered most possibly due to the enhancement in the side reactions discussed above ((12)–(17)), which lead to the undesired CH_4_, C_2_H_4_, and most possibly carbon formation, which eventually results in catalyst deactivation. The increase in $S_{CO}$ implies that either reaction (14) becomes significant and/or part of the coke formed was gasified via the Reverse Boudouard reaction (3). The deposition of carbon was corroborated by the TPO experiment conducted immediately after the stability test. The profile of the CO_2_ thus produced was qualitatively similar with that presented in Figure 10, with the amount of CO_2_ thus produced being significantly higher (6534.5 μmol·g^−1^) and accompanied by a parallel, but smaller, production of CO (94.4 μmol·g^−1^) when the reaction was taking place at 600 °C (Figure S9). The simultaneous consumption of CO_2_ above 750 °C, where CO was eluted, implies that part of the CO_2_ produced during TPO interacted with the accumulated carbon, generating CO most possibly through the reverse Boudouard reaction [64]. As it is observed in Figure S9, a significantly longer time (95 min) of stay at 800 °C was required in order for carbon to be completely removed from the catalyst surface compared to that shown in Figure 10b. Although catalytic activity was partially regained following the complete carbon oxidation during the TPO experiment, a similar loss of catalytic activity was observed after the subsequent catalyst exposure to the gas stream for 4 h (Figure S8b). The affinity of 30%Ga_2_O_3_-Al_2_O_3_ catalyst towards coke formation was most likely related to the large number of acidic sites characterized in this sample (Table 3, Figure 4) [2,28,65].

Catalyst was also deactivated when the TOS stability test was conducted at 650 °C, as shown by the substantial decrease in propane conversion from 45 to 5.5% after continuous catalyst operation for ~12 h (Figure S10a). Contrary to what was observed at 600 °C, $S_{C_{3}H_{6}}$ remained stable at 650 °C ranging between 38 and 42%, while $S_{CO}$ decreased from 35 to 22% and $S_{CH_{4}}$ and $S_{C_{2}H_{4}}$ increased from 8 to 14% and from 10 to 26%, respectively. These findings demonstrate that the formation of CH_4_ and C_2_H_4_ was enhanced with time at the expense of CO, implying that the side reactions ((12)–(17)), which produced C_2_H_4_, CH_4_ and C, inhibited the RWGS (2) and reverse Boudouard (3) reactions, which produced CO. It can then be suggested that the rate of carbon deposition was higher than the rate of carbon gasification through the reverse Boudouard (3) reaction when the reaction occurred at 650 °C. This was also confirmed by the TPO experiment (Figure S11) conducted after the TOS stability test shown in Figure S10a, where higher amounts of CO_2_ (6743.0 μmol·g^−1^) and CO (184.3 μmol·g^−1^) were produced. It should be noted that the catalyst needs to remain at 800 °C for 120 min in order for the oxidation of carbon to be completed. The subsequent exposure of the catalyst to CO_2_-ODP conditions showed that catalytic activity was restored following carbon oxidation, but it was rapidly lost within the first 5 h on stream.

The results discussed above clearly indicate that carbon deposition on the catalyst surface is enhanced as the reaction temperature increases, leading to gradual catalyst deactivation. However, catalytic activity remains stable under conditions (T < 600 °C) where the formation C_2_H_4_, CH_4_ and C_2_H_6_ is limited providing evidence that the 30%Ga_2_O_3_-Al_2_O_3_ is an efficient catalyst for the CO_2_-assisted hydrogenation of propane provided that the reaction conditions, and especially reaction temperature, are properly selected in order side reactions to be suppressed.

### 3.4. In Situ DRIFTS Studies for the CO_2_-ODP Reaction

**Figure 11:**
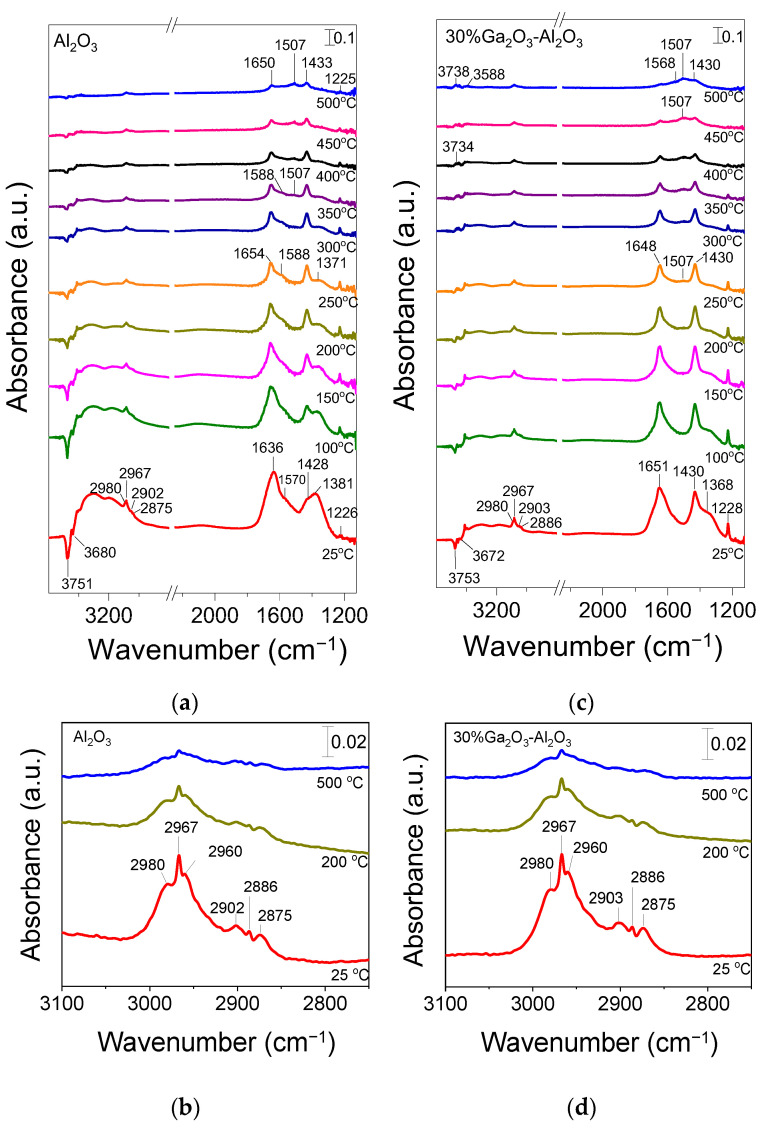
DRIFT spectra obtained over the (**a**) Al_2_O_3_ and (**c**) 30%Ga_2_O_3_-Al_2_O_3_ catalysts under 1% C_3_H_8_ + 5% CO_2_ (in He) flow in the temperature range of 25–500 °C. The corresponding DRIFT spectra obtained at 25, 200, and 500 °C in the 3100–2750 cm^−1^ region are presented in (**b**,**d**).

The CO_2_-ODP reaction was also investigated by in situ DRIFTS in an attempt to identify the reaction intermediates formed on the catalyst surface under reaction conditions. Representative DRIFT spectra collected at selected temperatures over bare Al_2_O_3_ and 30%Ga_2_O_3_-Al_2_O_3_ catalysts following their interaction with a feed stream of 1%C_3_H_8_ + 5%CO_2_ (in He) at 25 °C are presented in Figure 11. In the case of bare Al_2_O_3_ (Figure 11a), the spectrum recorded at 25 °C was consisted of two negative bands (3751 and 3680 cm^−1^) due to surface OH groups of Al_2_O_3_ which may serve as active sites for CO_2_ adsorption, two bands previously assigned to bicarbonate species (1428 and 1226 cm^−1^), three bands due to unidentate and bidentate carbonates (1636, 1570 and 1381 cm^−1^) and several bands in the *ν*(C-H) region (3000–2850 cm^−1^) [36,37,38,39,40,41,42]. It should be noted that the pair of peaks at 1570 and 1381 cm^−1^ is also characteristic of formate species and therefore, their formation on the catalyst surface cannot be excluded [3,66,67,68]. If this is the case, then the occurrence of the RWGS may be possible since formates have been proposed as active intermediates in this reaction [69]. Regarding the bands detected in the wavenumber range of 3000–2850 cm^−1^, they can be better seen in Figure 11b, where six bands can be clearly discerned which can be attributed to asymmetric (2980 and 2967 cm^−1^) and symmetric (2960 cm^−1^) C–H stretching vibrations in methyl groups (CH_3,ad_), to asymmetric (2902 cm^−1^) and symmetric (2875 cm^−1^) vibrations in methylene groups (CH_2,ad_) as well as to *ν*_s_(CH_2_)/ν_as_(CH_3_) of gaseous propane (2886 cm^−1^) [5,67,70,71].

Stepwise increase in temperature at 350 °C led to a gradual decrease in the relative intensity of all bands as well as to the splitting of the peak at 1570 cm^−1^ into two peaks located at 1588 and 1507 cm^−1^. As mentioned above, the former one was previously assigned to bidentate or formate species, while the latter one can be attributed to unidentate carbonates adsorbed on the Al_2_O_3_ surface [3,40,47,66,68,72,73]. It is of interest to note that a similar band detected at 1507 cm^−1^ under propane dehydrogenation conditions, both in the presence and absence of CO_2_ over Ga_2_O_3_-Al_2_O_3_ catalysts, was also attributed by Han et al. [3] to adsorbed C_3_H_7_* species, the dehydrogenation of which was found to be the rate-determining step. As can be seen in Figure 11a, a further increase in temperature to 500 °C resulted in an increase in the relative intensity of the band at 1507 cm^−1^, implying that the formation of the corresponding species was enhanced at elevated temperatures. This further supports the above suggestion that C_3_H_8_ dehydrogenation to adsorbed C_3_H_7_* species may take place under the present reaction conditions.

Similar peaks were detected in the DRIFT spectra obtained over the 30%Ga_2_O_3_-Al_2_O_3_ catalyst (Figure 11c,d), indicating that the addition of Ga_2_O_3_ did not affect the nature of the species formed under reaction conditions. The main difference observed was that the band at 1507 cm^−1^ started to be developed at lower temperatures (~250 °C) compared to bare Al_2_O_3_ and its relative intensity was found to be higher at a given temperature implying that the formation rate of the corresponding species was higher over the most active 30%Ga_2_O_3_-Al_2_O_3_ catalyst (Figure S12). This reinforces the above assumption that the band at 1507 cm^−1^ was due to adsorbed C_3_H_7_* species. Moreover, the relative intensity of the bands due to bicarbonates (1430 and 1228 cm^−1^) was higher in the presence of 30%Ga_2_O_3_ on Al_2_O_3_, especially below 250 °C (Figure 11), most possibly due to the higher basicity of this sample which enhanced the CO_2_ activation in agreement with the results of Figure S3. In addition to CO_2_ activation, the enhanced surface basicity has also been reported to hide the adsorption of the undesired C_2_H_4_ on the catalyst surface, thus inhibiting its subsequent deep oxidation to carbon oxides [74]. It should be noted that a new band at 3734 cm^−1^ was developed at ~400 °C, which, according to the literature, was due to surface OH groups created by H_2_O adsorption [3,5,75]. This band increased in intensity with increasing temperature up to 500 °C and was accompanied by the appearance of a new weak band at 3588 cm^−1^, which was previously suggested to be raised through H_2_O interaction with weak basic hydroxyl groups on the metal oxide surface [75]. Water adsorption may be generated through the RWGS reaction [3,5], which seems to be enhanced over the most active 30%Ga_2_O_3_-Al_2_O_3_ catalyst, as evidenced by the absence of similar bands from the spectra obtained from the least active Al_2_O_3_ support at least below 500 °C.

In an attempt to further explore the reactivity of surface species formed under CO_2_-ODP conditions, a DRIFTS experiment was conducted under transient conditions at constant temperature over the most active 30%Ga_2_O_3_-Al_2_O_3_ catalyst. In this experiment, the catalyst was exposed to 1%C_3_H_8_ + 5%CO_2_/He at 500 °C, followed by spectra recording as a function of time. As it can be seen in Figure S13, the spectrum collected at 2 min is characterized by bands due to (a) asymmetric (2982 and 2966 cm^−1^) C–H stretching vibrations in methyl groups (CH_3,ad_), (b) asymmetric (2903 cm^−1^) vibrations in methylene groups (CH_2,ad_), (c) bicarbonate species (1435 and 1228 cm^−1^), (d) bidentate carbonate species (1634 cm^−1^) and adsorbed C_3_H_7_* species (1505 cm^−1^). Stepwise increase in reaction time to 20 min resulted in a progressive decrease in the relative population of bicarbonates and bidentate carbonates, accompanied by an increase in that of C_3_H_7_* species and the gradual development of two new bands at 1588 and 1390 cm^−1^ due to formate formation on the catalyst surface (Figure S13b). Although the bands in the 3100–2750 cm^−1^ region remained unaffected with increasing the reaction time up to 20 min, a new band was discerned at 3085 cm^−1^ after 30 min on stream, which according to previous studies can be attributed to asymmetric vibrations of the C-H bond of methylene (CH_2,ad_) groups of adsorbed propylene on the catalyst surface (Figure S13a) [76]. Further increase in reaction time up to 60 min led to an additional increase in the intensity of bands due to formates, C_3_H_7_* species and adsorbed propylene and the development of two shoulders at 2935 and 2921 cm^−1^ which can be assigned to *ν_as_*(CH_3_) and *ν_s_*(CH_3_) of adsorbed propylene [76]. Results of Figure S13 clearly indicate that both the RWGS and propane dehydrogenation reactions are operable at 500 °C over the 30%Ga_2_O_3_-Al_2_O_3_ catalyst.

In order to corroborate the contribution of adsorbed and/or gas phase propylene to the bands detected under CO_2_-ODP conditions, an additional DRIFTS experiment was carried out where the interaction of the 30%Ga_2_O_3_-Al_2_O_3_ catalyst with a 10% C_3_H_6_ (in He) mixture was investigated in the temperature range of 25–500 °C. Results (Figure S14) showed that propylene adsorption led to the appearance of several spectral features in the *ν*(C-H) region which were due to asymmetric and symmetric C-H bond vibrations of the methyl (CH_3,ad_) and methylene (CH_2,ad_) groups of adsorbed or gas phase propylene [76,77]. The bands at 3085, 2935, and 2921 cm^−^^1^ observed in Figure S13a are also discernible in Figure S14b, confirming the production of propylene when the catalyst interacts with the 1%C_3_H_8_ + 5%CO_2_ (in He) mixture. Six bands were also observed below 1700 cm^−1^ (Figure S14a) which were attributed to the C=C bond stretch (1665 and 1638 cm^−1^) as well as to asymmetric and symmetric bending vibrations of the methyl (CH_3,ad_) groups (1475, 1442, 1393, 1377 cm^−1^) of adsorbed or gas phase propylene [76,77]. Taking into account that some of these features were also present in the spectra of both Figure 11 and Figure S13, it can be argued that generated propylene may also contribute to their development.

Based on the above, it can be suggested that both C_3_H_8_ and CO_2_ are activated on the catalyst surface as evidenced by the formation of CH_x_ and carbonate-like species, respectively, at low reaction temperatures. Regarding the CO_2_-ODP reaction mechanism, two general mechanistic schemes have been proposed: the one-step oxidative route and the two-step oxidative route, which differ mainly in the role of CO_2_ [3,5,18]. According to the former one, the lattice oxygen ions abstract hydrogen atoms from C_3_H_8,_ producing C_3_H_6_ and H_2_O, while CO_2_ re-oxidizes the reduced surface following the Mars–Van Krevelen mechanism to complete the redox cycle [18]. In order to explore if the catalyst surface is able to easily re-oxidized by CO_2_, immediately after the H_2_-TPR experiment conducted over the 30%Ga_2_O_3_-Al_2_O_3_ catalyst (Figure S6), the flow was switched to a 5%CO_2_/He mixture at 500 °C for 30 min followed by a subsequent H_2_-TPR under identical conditions with those discussed above. No reduction peak was observed in the H_2_-TPR profile (Figure S15), implying the CO_2_ was not able to re-oxidize the catalyst surface, providing additional evidence that the one-step oxidative route was not operable for the Ga_2_O_3_-Al_2_O_3_ catalysts of the present study. Results are in agreement with those reported by Getsoian et al. [13], who demonstrated that Ga^3+^ was not reduced to Ga^+^ over Ga–SiO_2_ and Ga–H-BEA catalysts rendering the redox mechanism unfavorable and was also supported by computational studies over Ga-zeolite catalysts, which demonstrated that non-redox mechanisms of alkane dehydrogenation reactions proceed with much lower energy barriers than those required for the reduction of Ga^3+^ to Ga^+^ [78,79].

Based on the DRIFTS results of Figure 11 and Figures S12–S14, the reaction seems to proceed via the two-step oxidative route, according to which C_3_H_8_ is dehydrogenated on the catalyst’s acid sites, leading to the formation of an intermediate adsorbed surface C_3_H_7_* species. Hydrogen produced from this step is removed with the indirect contribution of CO_2,_ which is activated on the catalyst’s basic sites and participates in the RWGS reaction, shifting the thermodynamic equilibrium towards C_3_H_6_ formation. The RWGS, which has been proposed to occur via intermediate formation of formate species (originating by carbonates/bicarbonates interaction with hydrogen atoms [80,81,82]), is operable under the present reaction conditions as evidenced by the detection of bands due to adsorbed formates and steam and seems to be enhanced in the presence of Ga_2_O_3_. The enhancement in the RWGS may also be responsible for the inhibition of the C–C bond cleavage of the intermediate C_3_H_7_* species, thus leading to a decrease in the formation rates of the undesired C_2_H_x_ and CH_4_ and their corresponding selectivities (Figure 4d and Figure S7). The above findings demonstrate that although CO_2_ does not directly participate in the dehydrogenation step, its role is decisive in propylene production. Results of the present study clearly show that the aforementioned steps of the C_3_H_7_* formation and the RWGS reaction are favored over the 30%Ga_2_O_3_-Al_2_O_3_ catalyst characterized by a moderate number and strength of both acid and basic sites, confirming the crucial role of acid/base properties on propylene production.

### 3.5. CO_2_-ODP Reaction Scheme Under Transient Conditions

**Figure 12:**
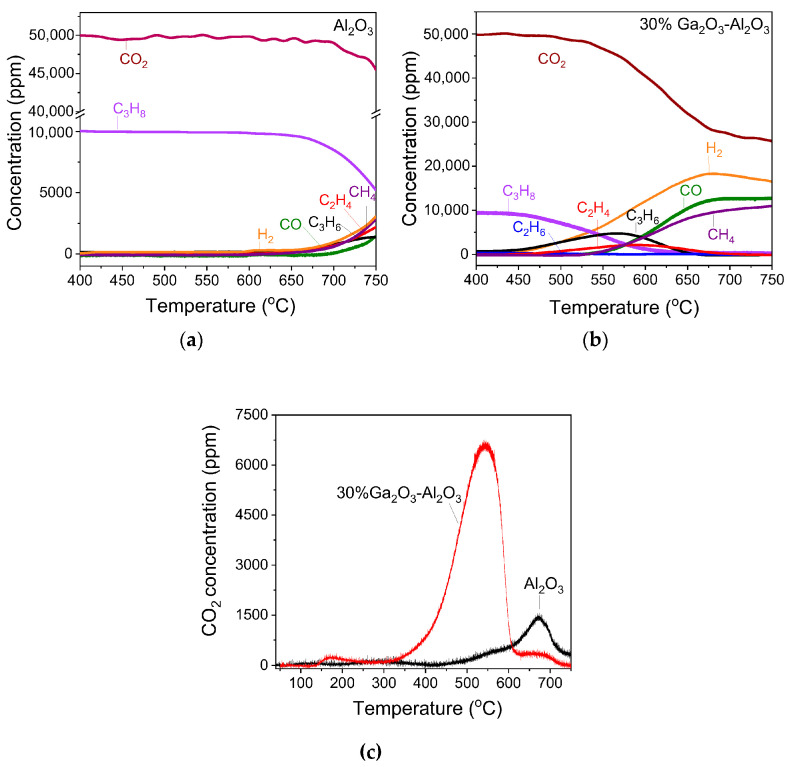
Transient-MS spectra obtained over the (**a**) Al_2_O_3_ and (**b**) 30%Ga_2_O_3_-Al_2_O_3_ catalysts following interaction with the reaction mixture 1% C_3_H_8_ + 5% CO_2_ (in He) at 25 °C and subsequent linear heating at 750 °C (*β* = 10 °C/min). (**c**) Responses of CO_2_ produced during TPO with 1% O_2_ (in He) occurred immediately after the TPSR experiments of (**a**,**b**).

The reaction scheme was also investigated by transient-MS technique over bare Al_2_O_3_ and 30%Ga_2_O_3_-Al_2_O_3_ catalysts using a feed composition consisting of 1%C_3_H_8_ + 5% CO_2_ (in He) and a linear temperature ramp of 10 °C/min. The TPSR profile obtained from bare Al_2_O_3_ is shown in Figure 12a, where it is observed that the concentrations of reactants, CO_2_ and C_3_H_8_, started to decrease at temperatures higher than 650 °C. This decrease was accompanied by the simultaneous evolution of C_3_H_6_, CO, H_2_, CH_4_, and C_2_H_4,_ implying that in addition to the propane oxidative dehydrogenation, the reactions of propane and propylene decomposition and/or propane hydrogenolysis ((12)–(17)) were taking place. The concentrations of CH_4_ and C_2_H_4_ became higher than that of C_3_H_6_ above 720 °C, implying that the latter undesired reactions were favored with increasing temperature, in excellent agreement with the results of catalytic performance tests (Figure 6a).

The TPSR pattern obtained from 30%Ga_2_O_3_-Al_2_O_3_ catalyst is presented in Figure 12b where it can be seen that propane dehydrogenation was initiated at significantly lower temperatures compared to bare Al_2_O_3_, as evidenced by the onset of C_3_H_8_ consumption and C_3_H_6_ and H_2_ evolution at ~450 °C, i.e., at temperatures where the FTIR band assigned to adsorbed C_3_H_7_* species (1507 cm^−1^) was clearly discerned (Figure 11b). The concentration of C_3_H_6_ went through a maximum at around 570 °C and then progressively decreased with further increasing temperature, in excellent agreement with the results of Figure 5b, where $Y_{C_{3}H_{6}}$ was found to be optimized at ~600 °C. As noted above, the decrease in C_3_H_6_ concentration can be attributed to its consumption via the propylene decomposition reaction (13), which may be responsible for the evolution of CH_4_ and C_2_H_4_. Contrary to C_3_H_6_, the hydrogen concentration was progressively increased with increasing temperature up to 680 °C and was slightly decreased when the temperature reached 750 °C. This indicates that the origin of H_2_ generation was not limited to propane dehydrogenation reaction, but as discussed above it may be also produced through the propane decomposition (16) and the dry reforming of propane (18), with the former reaction being more possible taking into account that H_2_ concentration is twice that of CH_4_ (Figure 12b). This was further supported by the fact that although C_2_H_4_ was eliminated above 670 °C, CH_4_ concentration increased continuously with increasing temperature to 750 °C.

Propane hydrogenolysis via reactions (12) and (17) may also contribute to the continuous upward trend of CH_4_ response, as well as to the observed production of C_2_H_6_ traces (reaction (12)). The concentration of CO_2_ started to decrease at similar temperatures with C_3_H_8_ (~450 °C), and as discussed above, it can be converted to CO via the RWGS (2), the reverse Boudouard (3), and/or the dry reforming of propane (18). However, CO started to elute above 550 °C. This can be correlated with DRIFTS results of Figure 11c, where it was shown that CO_2_ was initially (at low reaction temperatures) adsorbed on the catalyst surface in the form of carbonate-like species which most possibly interact with hydrogen atoms that were abstracted from propane molecule during its dehydrogenation, yielding formates (RWGS reaction), which are considered, at least in part, as precursors of CO (generated at higher reaction temperatures). Moreover, the formation of carbonate-like structures was previously found to be advantageous for the reaction between CO_2_ and coke during the reverse Boudouard reaction pathway [83].

Immediately after completion of the TPSR experiments presented in Figure 12a,b, TPO experiments were conducted in order to estimate the amount of carbon deposited on the catalyst surface during CO_2_-ODP reaction. The profile of CO_2_ (Figure 12c) thus produced from the bare Al_2_O_3_ sample exhibited a weak peak at 315 °C as well as a shoulder at around 550 °C followed by a peak of higher intensity centered at around 675 °C, indicating that three distinct carbon species are present on the surface of “spent” Al_2_O_3_ which can be attributed to the propylene and/or propane decomposition reactions ((13) and (16)). This was also the case for the 30%Ga_2_O_3_-Al_2_O_3_ catalyst, the CO_2_ response curve of which consisted of two weak peaks at 175 and 670 °C and a major one with a maximum at 545 °C. The amounts of CO_2_ produced during TPO experiments, which is equivalent to the amount of carbon deposited during TPSR experiments, were estimated by integrating the area below the CO_2_ response curves and found to be significantly higher for the 30%Ga_2_O_3_-Al_2_O_3_ catalyst (496.5 μmol g^−1^ corresponding to 9.6 μmol m^−2^) than bare Al_2_O_3_ (115.8 μmol g^−1^ corresponding to 1.8 μmol m^−2^). This may be due to the higher acid site density of the 30%Ga_2_O_3_-Al_2_O_3_ catalyst (Table 3), which has been previously accused of the enhanced tendency of the catalyst towards carbon deposition [2,8,14,17,84] and is most possibly correlated with the short lifetime of catalyst at reaction temperatures higher than 600 °C (Figures S8 and S10). It should be mentioned that despite the higher amount of carbon deposition on the 30%Ga_2_O_3_-Al_2_O_3_ surface, the evolution of the CO_2_ peaks appeared at lower temperatures for this sample, implying that coke gasification is facilitated compared to bare Al_2_O_3_.

The overall carbon balance of the TPSR experiments was calculated using the following equation, where the amount of coke formed on the catalyst surface estimated by TPO experiments was taken into account as follows:

(19) $${\left[Carbon\;total\right]}_{total}=\frac{\left[CO\right]+\left[CO_{2}\right]+\left[CH_{4}\right]}{3}+2\cdot \frac{\left[C_{2}H_{4}\right]+\left[C_{2}H_{6}\right]}{3}+\left[C_{3}H_{8}\right]+\left[C_{3}H_{6}\right]+\left[C_{accumulated}\right]$$

For both catalysts examined, the carbon balance was satisfactory, with a deviation of 1% for Al_2_O_3_ and 5% for 30%Ga_2_O_3_-Al_2_O_3_.

Overall, it can be suggested that the dispersion of a suitable amount of gallium oxide on the alumina surface is able to modify the acid/base properties of the alumina support, providing the appropriate number of both acidic and basic sites. This facilitates the activation of reactants and their selective conversion to C_3_H_6_ and CO at low temperatures, where undesired reactions are suppressed, ensuring a stable catalyst performance with time on stream.

## 4. Conclusions

The effect of Ga_2_O_3_ content on the physicochemical properties of alumina-supported catalysts and their catalytic performance for the CO_2_-assisted oxidative dehydrogenation of propane was reported herein, aiming to optimize catalyst composition. Results obtained are summarized as follows:

1. The role of Ga_2_O_3_ loading is to provide a suitable number and strength of acidic and basic sites that are able to effectively activate the reactants and suppress the side reactions, thus ensuring high propylene yields.
2. Catalytic activity was found to be strongly influenced by the Ga_2_O_3_ concentration and optimized for the 30%Ga_2_O_3_-Al_2_O_3_ catalyst, which was characterized by moderate surface acidity and basicity. This catalyst was not only able to enhance the propane conversion to propylene which reached 59% at ~600 °C with a corresponding propylene yield of 39%, but also to limit the undesired reactions of propane hydrogenolysis and propane/propylene decomposition which were responsible for the formation of C_2_H_4_, CH_4_, C_2_H_6_ and coke.
3. The variation in the $S_{C_{3}H_{6}}/S_{C_{2}H_{4}}$ ratio with the acid/base properties provided evidence that the C–H bond cleavage was facilitated compared to the C–C bond break over samples characterized by moderate surface basicity and acidity.
4. The TOS tests followed by TPO experiments showed that coke formation was favored with increasing reaction temperature over 30%Ga_2_O_3_-Al_2_O_3_ catalyst, leading to progressive catalyst deactivation when the reaction was taking place at temperatures higher than 600 °C, which, however, can be completely (at 650 °C) or partially (at 600 °C) restored by subsequent oxidation of carbon. However, the conduction of CO_2_-ODP reaction at 550 °C led to a higher propylene selectivity, lower carbon formation, and very good stability with time on stream, indicating the potential suitability of the 30%Ga_2_O_3_-Al_2_O_3_ catalyst in the CO_2_-assisted hydrogenation of propane at temperatures of practical interest.
5. Results of TPSR and DRIFTS experiments indicated that the reaction proceeds through a two-step oxidative route which includes the dehydrogenation of propane on the catalyst’s acid sites towards the formation of an intermediated adsorbed C_3_H_7_* species and hydrogen atoms, which are abstracted by CO_2_ adsorbed on the catalyst’s basic sites and converted to formates and, eventually, CO via the RWGS reaction. Although carbon deposition was favored on the 30%Ga_2_O_3_-Al_2_O_3_ surface compared to bare Al_2_O_3_, CO_2_ was eluted at lower temperatures during TPO experiments, implying that coke gasification is facilitated in the presence of Ga_2_O_3_.

## Figures and Tables

**Table 1:** BET specific surface area of Ga_2_O_3_-Al_2_O_3_ catalysts.

Catalyst	Specific Surface Area (m^2^ g^−1^)
Al_2_O_3_	64.4
10%Ga_2_O_3_-Al_2_O_3_	59.0
20%Ga_2_O_3_-Al_2_O_3_	53.0
30%Ga_2_O_3_-Al_2_O_3_	51.6
40%Ga_2_O_3_-Al_2_O_3_	46.2
Ga_2_O_3_	4.0

**Table 2:** Amount of desorbed CO_2_ during CO_2_-TPD experiments.

Catalyst	LT Peak (μmol m^−2^)	HT Peak (μmol m^−2^)	Total Amount of Desorbed CO_2_ (μmol m^−2^)
Al_2_O_3_	0.48	0.06	0.54
10%Ga_2_O_3_-Al_2_O_3_	0.82	0.01	0.83
20%Ga_2_O_3_-Al_2_O_3_	1.09	0.45	1.54
30%Ga_2_O_3_-Al_2_O_3_	1.03	0.26	1.29
40%Ga_2_O_3_-Al_2_O_3_	0.90	0.18	1.08
Ga_2_O_3_	0.03	0.72	0.75

**Table 3:** Surface acidity of the synthesized Ga_2_O_3_-Al_2_O_3_ catalysts estimated by potentiometric titration experiments.

Catalyst	Acid Sites Density (μmol·m^−2^)			
	A_vw_ (Very Weak)	A_w_ (Weak)	A_s_ (Strong)	A_total_
Al_2_O_3_	0.39	3.91	2.34	6.64
10%Ga_2_O_3_-Al_2_O_3_	0.47	2.66	5.59	8.75
20%Ga_2_O_3_-Al_2_O_3_	0.40	3.02	5.85	9.26
30%Ga_2_O_3_-Al_2_O_3_	0.62	3.29	7.50	11.41
40%Ga_2_O_3_-Al_2_O_3_	0.52	4.26	5.76	10.54
Ga_2_O_3_	-	13.00	72.00	85.00

## Data Availability

Data are contained within the article and Supplementary Materials.

## Supplementary Materials

The following supporting information can be downloaded at https://www.mdpi.com/article/10.3390/nano15131029/s1. Table S1: Amount of desorbed CO_2_ during CO_2_-TPD experiments.; Table S2. Surface acidity of the synthesized Ga_2_O_3_-Al_2_O_3_ catalysts estimated by potentiometric titration experiments; Figure S1. (a) SEM images with (b) element mapping of Ga and (c) EDS profile obtained from the 10%Ga_2_O_3_-Al_2_O_3_ catalyst; Figure S2. TEM images and the corresponding SAED patterns acquired from the area denoted by the dashed lines obtained from (a, b) the bare Al_2_O_3_ and (c, d) the 10%Ga_2_O_3_-Al_2_O_3_; Figure S3. DRIFT spectra obtained from (A) Al_2_O_3_, (B) 10%Ga_2_O_3_-Al_2_O_3_, (C) 20%Ga_2_O_3_-Al_2_O_3_, (D) 30%Ga_2_O_3_-Al_2_O_3_, (E) 40%Ga_2_O_3_-Al_2_O_3_, and (F) Ga_2_O_3_ catalysts following adsorption of CO_2_ at 25 °C for 30 min and subsequent stepwise heating at the indicated temperatures under He flow; Figure S4. Potentiometric titration curves of the x%Ga_2_O_3_-Al_2_O_3_ catalyst suspensions in 0.05 M KNO_3_ and the corresponding Gran’s functions; (A) Control sample (without catalyst), (B) Al_2_O_3_, (C) 10%Ga_2_O_3_-Al_2_O_3_, (D) 20%Ga_2_O_3_-Al_2_O_3_ (E) 30%Ga_2_O_3_-Al_2_O_3_, (F) 40%Ga_2_O_3_-Al_2_O_3_, (G) Ga_2_O_3_; Figure S5. Effect of Ga_2_O_3_ content on the density of the different types of acid sites of x%Ga_2_O_3_-Al_2_O_3_ (x: 0–100 wt.%) catalysts; Figure S6. H_2_-TPR profiles obtained from the 10%Ga_2_O_3_-Al_2_O_3_ and 30%Ga_2_O_3_-Al_2_O_3_ catalysts; Figure S7. Ratio of propylene selectivity to ethylene selectivity at 600 °C as a function of the (A) Ga_2_O_3_ content, (B) total amount of desorbed CO_2_ during CO_2_-TPD experiments, and (C) the acid site density obtained over Al_2_O_3_, Ga_2_O_3_, and x%Ga_2_O_3_-Al_2_O_3_ catalysts; Figure S8. TOS stability test conducted at 600 °C under CO_2_-ODP conditions over the (A) fresh and (B) spent 30%Ga_2_O_3_-Al_2_O_3_ catalyst following TPO experiment; Figure S9. (A) Responses of CO_2_ and CO produced during the TPO experiment occurred after the TOS stability tests conducted at 600 °C, as presented in Figure S8a over the 30%Ga_2_O_3_-Al_2_O_3_ catalyst. In (B), the CO_2_ response at 800 °C was recorded as a function of time until complete oxidation of carbon; Figure S10. TOS stability test conducted at 650 °C under CO_2_-ODP conditions over the (A) fresh and (B) spent 30%Ga_2_O_3_-Al_2_O_3_ catalyst following TPO experiment; Figure S11. (A) Responses of CO_2_ and CO produced during the TPO experiment occurred after the TOS stability tests conducted at 650 °C, as presented in Figure S10a over the 30%Ga_2_O_3_-Al_2_O_3_ catalyst. In (B), the CO_2_ response at 800 °C was recorded as a function of time until complete oxidation of carbon; Figure S12. DRIFT spectra obtained at 350 and 500 °C in the 1900–1100 cm^−1^ region from the (A) Al_2_O_3_ and (B) 30%Ga_2_O_3_-Al_2_O_3_ catalysts under 1% C_3_H_8_ + 5% CO_2_ (in He) flow; Figure S13. DRIFT spectra obtained as a function of time at 500 °C in the (A) 3175–2725 cm^−1^ and (B) 1900–1100 cm^−1^ regions following interaction of 30%Ga_2_O_3_-Al_2_O_3_ catalysts with 1% C_3_H_8_ + 5% CO_2_ (in He); Figure S14. (A) DRIFT spectra obtained following interaction of the 30%Ga_2_O_3_-Al_2_O_3_ catalyst with 10% C_3_H_6_ (in He) in the temperature range of 25–500 °C. The corresponding DRIFT spectra obtained at 25, 200, and 500 °C in the 3100–2750 cm^−1^ region are presented in (B); Figure S15. H_2_-TPR profiles obtained from the 30%Ga_2_O_3_-Al_2_O_3_ catalyst after pre-oxidation with 5%O_2_/He and 5%CO_2_/He.
