# Supplementary material for: Effect of Ga2O3 Content on the Activity of Al2O3-Supported Catalysts for the CO2-Assisted Oxidative Dehydrogenation of Propane
Source: Nanomaterials (Basel). 2025 Jul 2;15(13):1029. doi: 10.3390/nano15131029 (PMC12251199; doi:10.3390/nano15131029)
Supplement: Supplementary file 1 [file nanomaterials-15-01029-s001.zip › nanomaterials-3693320-supplementary.pdf]

SUPPLEMENTARY MATERIAL

Effect of Ga_2O_3 content on the activity of Al_2O_3-supported catalysts for the CO_2-assisted oxidative dehydrogenation of propane

Alexandra Florou[1], Georgios Bampos[2], Panagiota D. Natsi[2], Aliki Kokka[1] and Paraskevi Panagiotopoulou[1,3*]

[1] Laboratory of Environmental Catalysis, School of Chemical and Environmental Engineering, Technical University of Crete, GR-73100 Chania, Greece

[2]Department of Chemical Engineering, University of Patras, GR-26504 Patras, Greece

[3] Institute of Geoenergy, Foundation for Research and Technology-Hellas (IG/FORTH), G-73100 Chania, Greece

* Correspondence: ppanagiotopoulou@tuc.gr; Tel.: +30-28210-37770

Table S1. Amount of desorbed CO_2 during CO_2-TPD experiments.

Catalyst	LT peak (μmol g^{-1})	HT peak (μmol g^{-1})	Total amount of desorbed CO_2 (μmol g^{-1})
Al_2O_3	31.0	4.0	35.0
10%Ga_2O_3-Al_2O_3	48.4	0.6	49.0
20%Ga_2O_3-Al_2O_3	57.6	23.8	81.4
30%Ga_2O_3-Al_2O_3	53.1	13.3	66.4
40%Ga_2O_3-Al_2O_3	41.5	8.2	49.7
Ga_2O_3	0.1	2.9	3.0

Table S2. Surface acidity of the synthesized Ga_2O_3-Al_2O_3 catalysts estimated by potentiometric titration experiments.

Catalyst	Acid Sites Density (μmol·g^{-1})			
	A_{vw} (Very weak)	A_w (Weak)	A_s (Strong)	A_{total}
Al_2O_3	25	252	151	428
10%Ga_2O_3-Al_2O_3	28	157	330	516
20%Ga_2O_3-Al_2O_3	21	160	310	491
30%Ga_2O_3-Al_2O_3	32	170	387	589
40%Ga_2O_3-Al_2O_3	24	197	266	487
Ga_2O_3	-	52	288	340

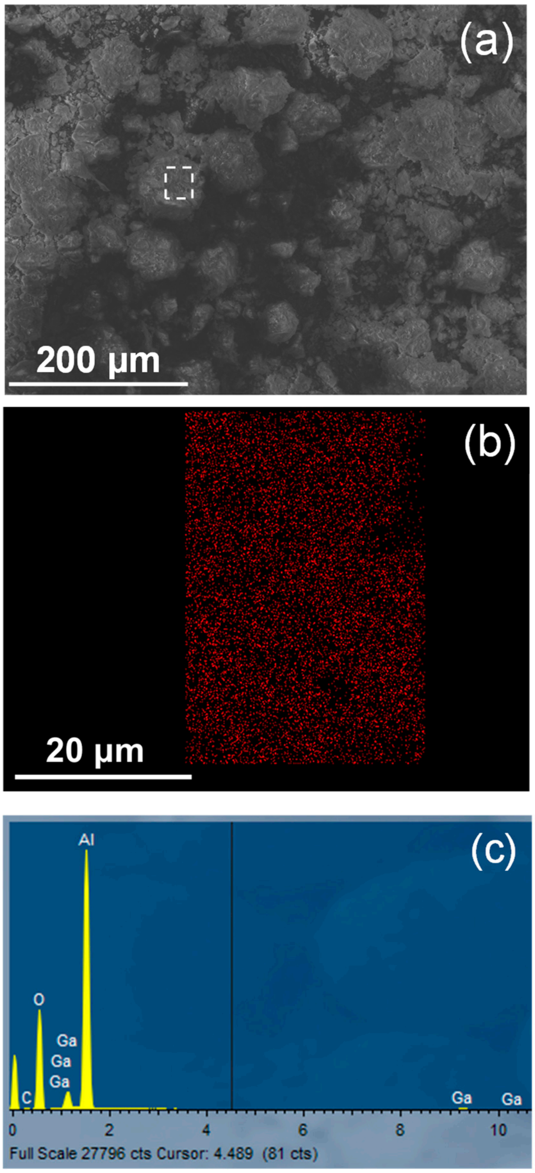

Figure S1. (a) SEM images with (b) element mapping of Ga and (c) EDS profile obtained from the 10% Ga_2O_3-Al_2O_3 catalyst.

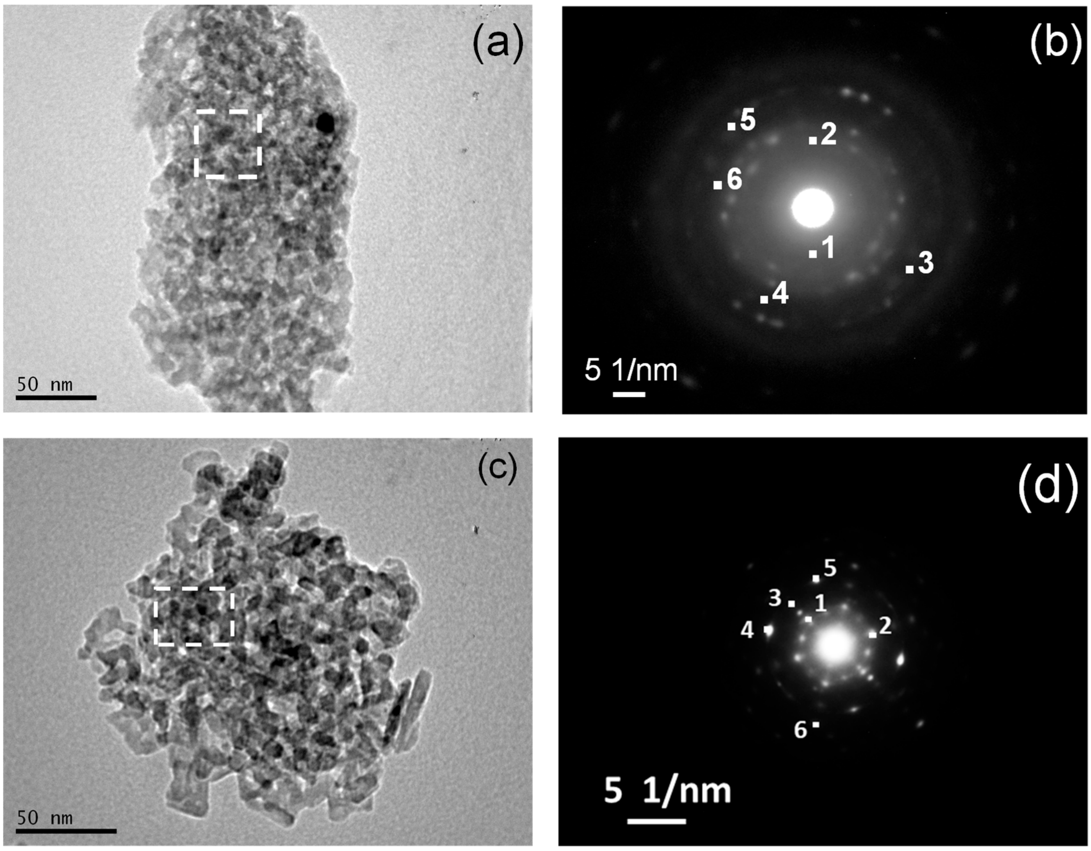

Figure S2. TEM images and the corresponding SAED patterns acquired from the area denoted by the dashed lines obtained from (a, b) the bare Al_2O_3 and (c, d) the 10% Ga_2O_3-Al_2O_3.

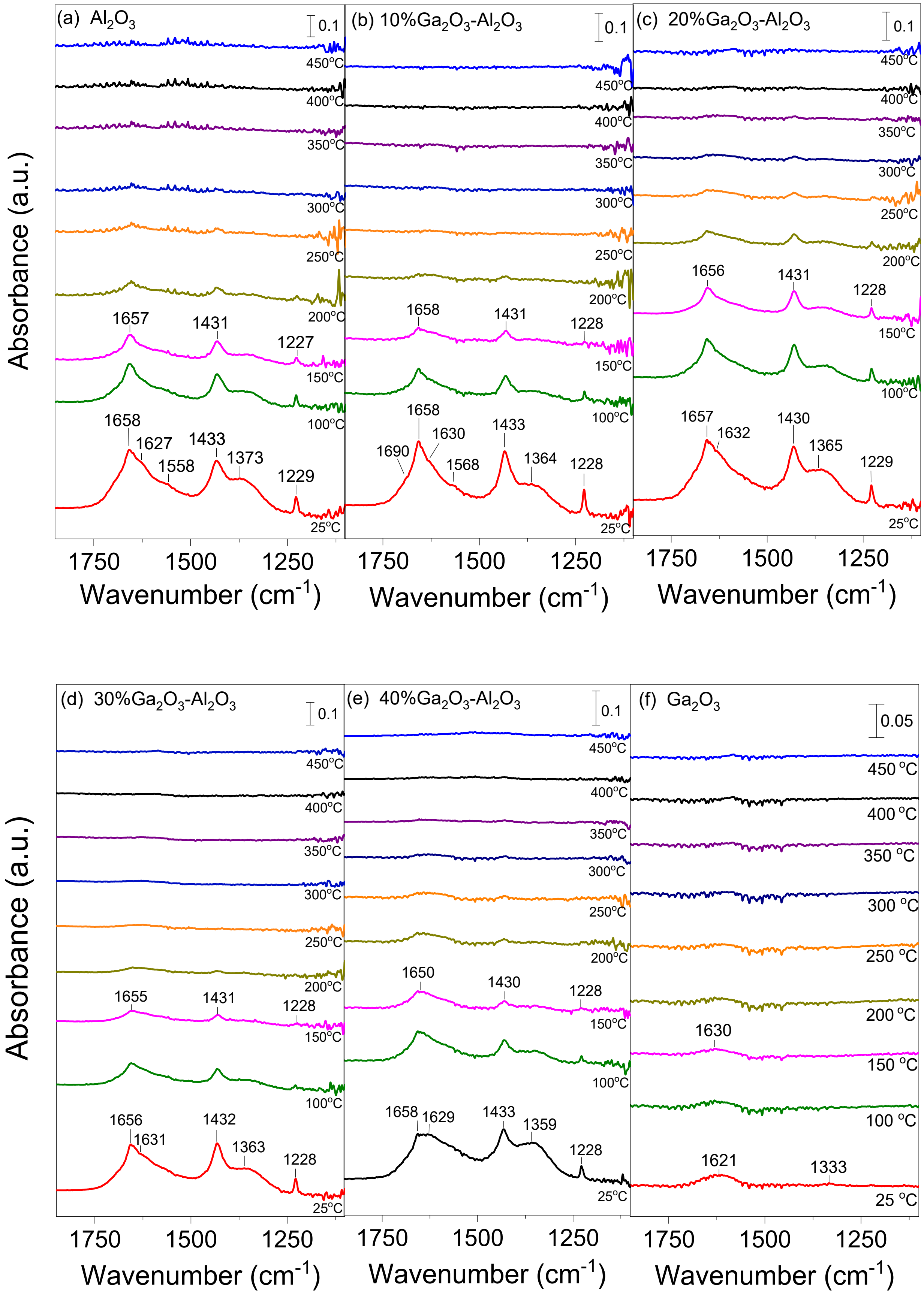

Figure S3. DRIFT spectra obtained from (a) Al_2O_3, (b) 10%Ga_2O_3-Al_2O_3, (c) 20%Ga_2O_3-Al_2O_3, (d) 30%Ga_2O_3-Al_2O_3, (e) 40%Ga_2O_3-Al_2O_3 and (f) Ga_2O_3 catalysts following adsorption of CO_2 at 25 °C for 30 min and subsequent stepwise heating at the indicated temperatures under He flow.

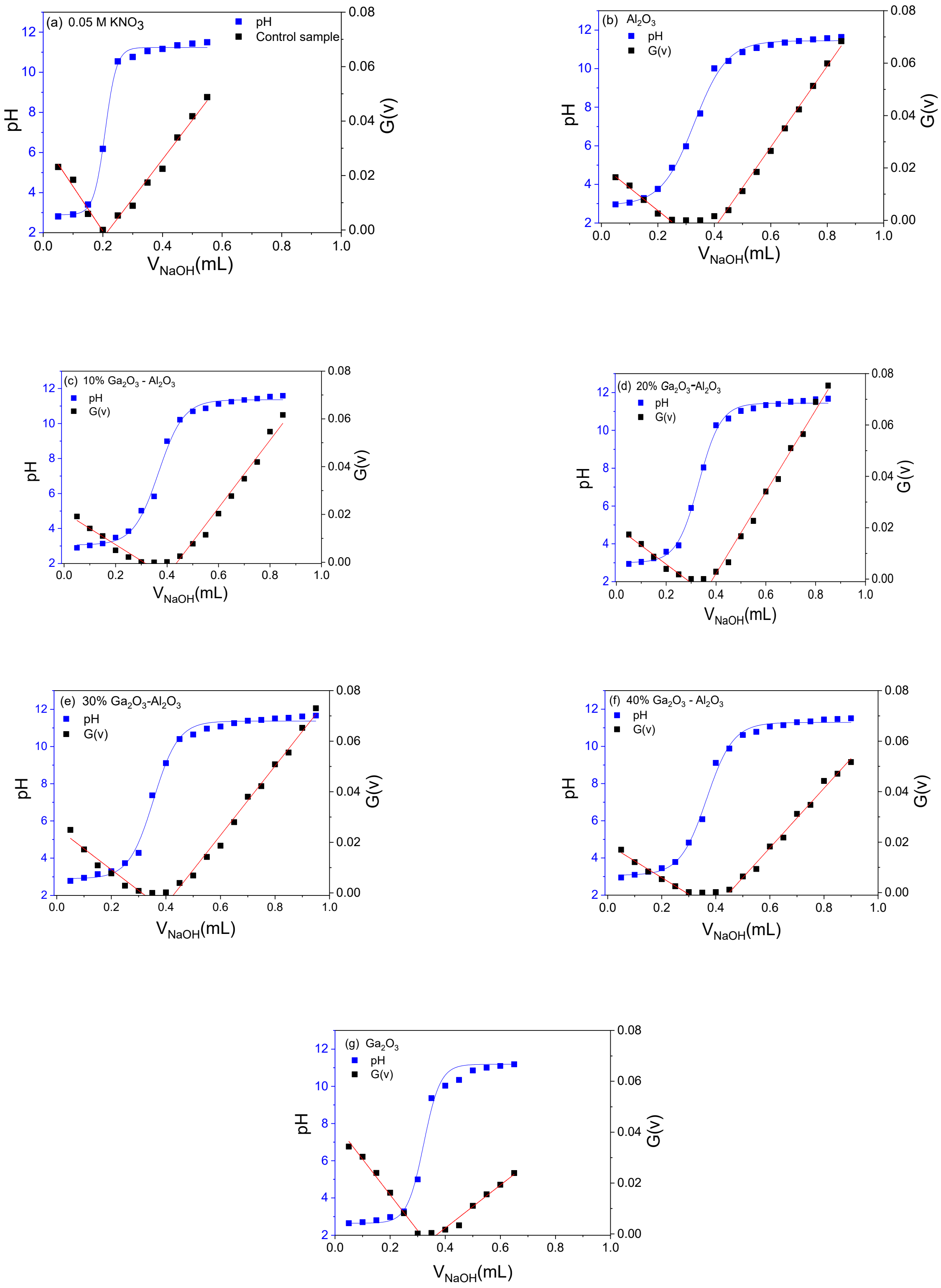

Figure S4. Potentiometric titration curves of the x%Ga_2O_3-Al_2O_3 catalyst suspensions in 0.05 M KNO_3 and the corresponding Gran's functions; (a) Control sample (without catalyst), (b) Al_2O_3, (c) 10%Ga_2O_3-Al_2O_3, (d) 20%Ga_2O_3- Al_2O_3 (e) 30%Ga_2O_3- Al_2O_3, (f) 40%Ga_2O_3- Al_2O_3, (g) Ga_2O_3.

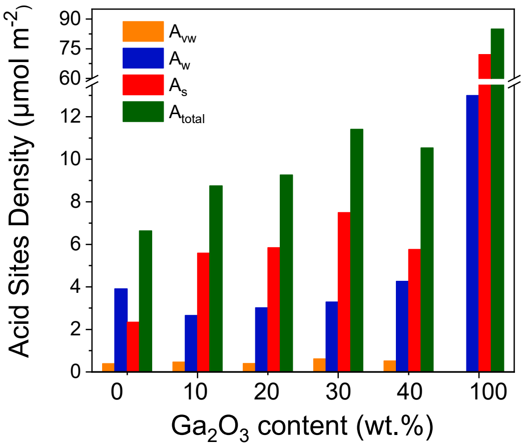

Figure S5. Effect of Ga_2O_3 content on the density of the different types of acid sites of x%Ga_2O_3-Al_2O_3 catalysts.

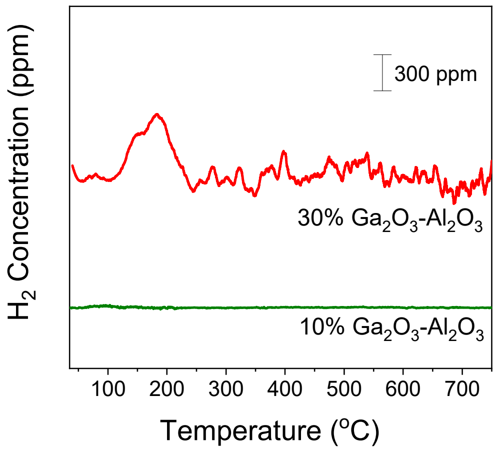

Figure S6. H_2-TPR profiles obtained from the 10%Ga_2O_3-Al_2O_3 and 30%Ga_2O_3-Al_2O_3 catalysts.

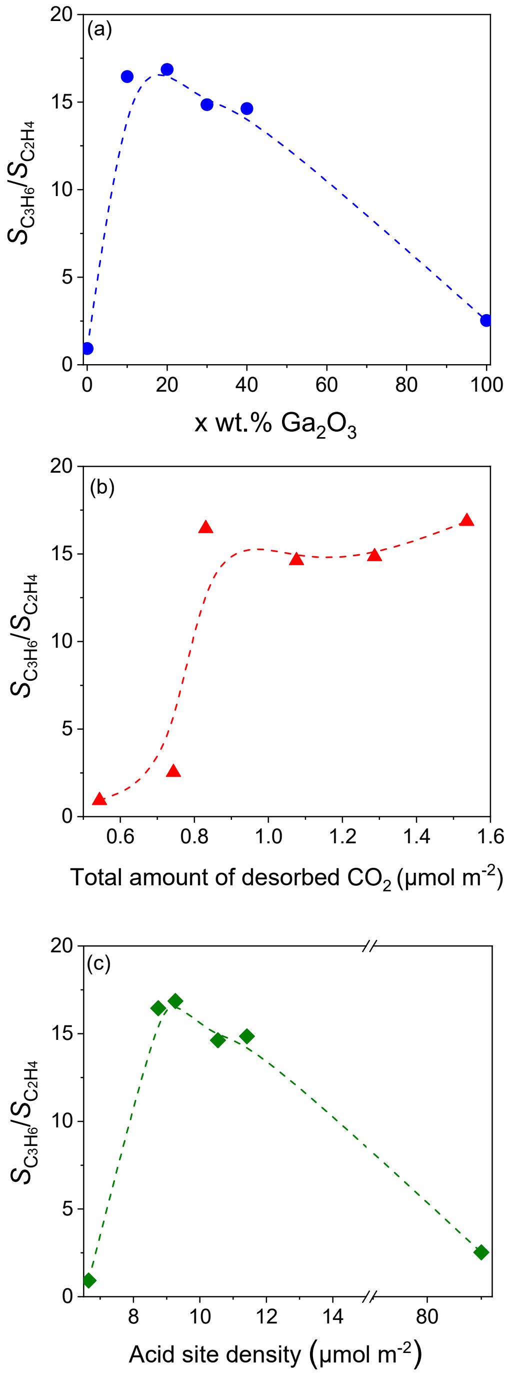

Figure S7. Ratio of propylene selectivity to ethylene selectivity at 600 °C as a function of the (a) Ga_2O_3 content, (b) total amount of desorbed CO_2 during CO_2-TPD experiments and (c) the acid site density obtained over Al_2O_3, Ga_2O_3 and x%Ga_2O_3-Al_2O_3 catalysts.

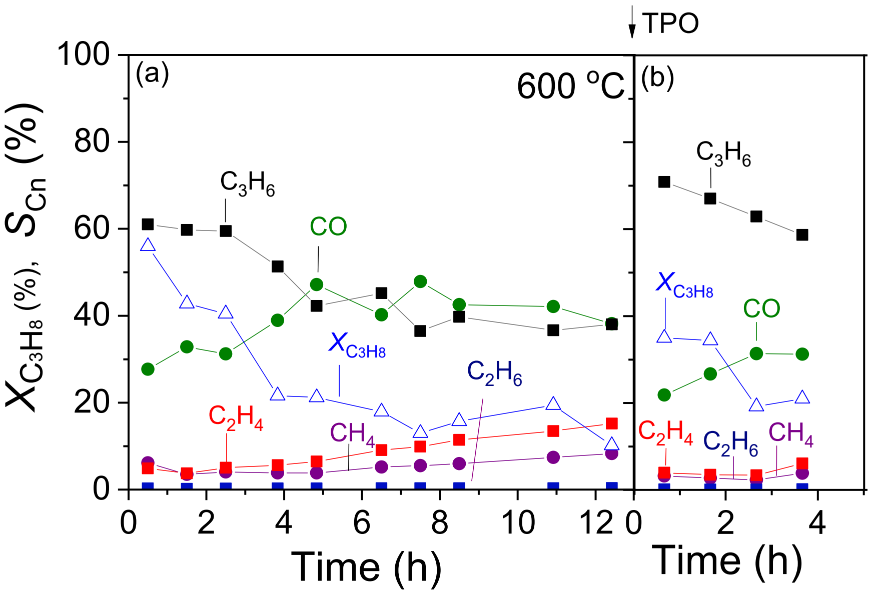

Figure S8. TOS stability test conducted at 600 °C under CO_2-ODP conditions over the (a) fresh and (b) spent 30%Ga_2O_3-Al_2O_3 catalyst following TPO experiment.

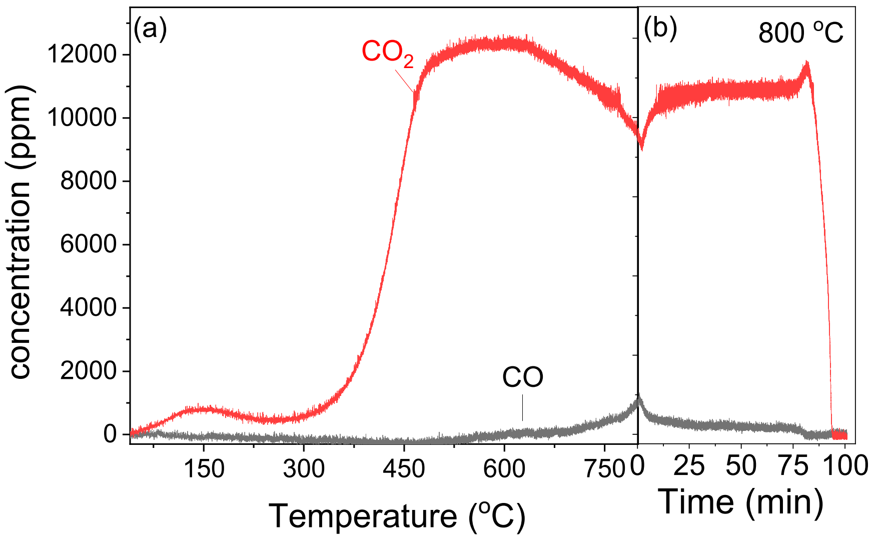

Figure S9. (a) Responses of CO_2 and CO produced during TPO experiment occurred after the TOS stability tests conducted at 600 °C presented in Fig.S4a over the 30% Ga_2O_3-Al_2O_3 catalyst. In (b), CO_2 response at 800 °C were recorded as a function of time until complete oxidation of carbon.

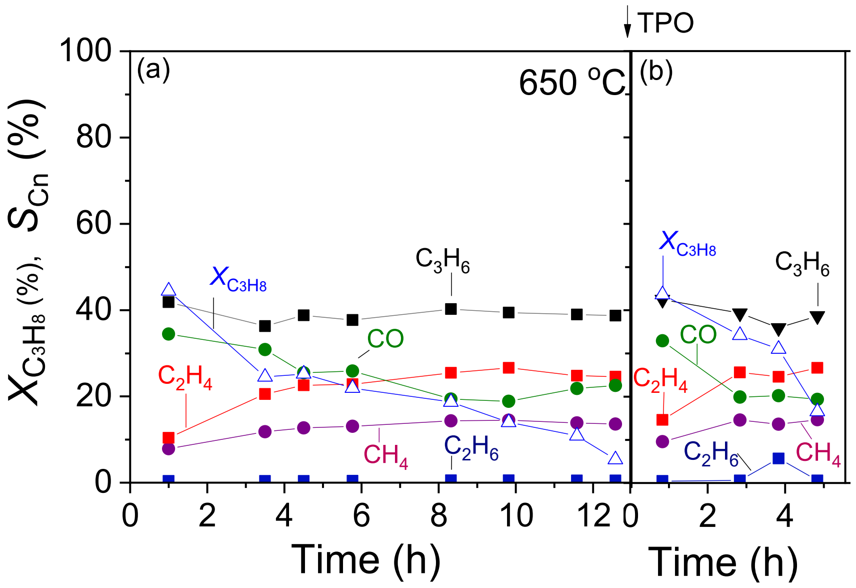

Figure S10. TOS stability test conducted at 650 °C under CO_2-ODP conditions over the (a) fresh and (b) spent 30%Ga_2O_3-Al_2O_3 catalyst following TPO experiment.

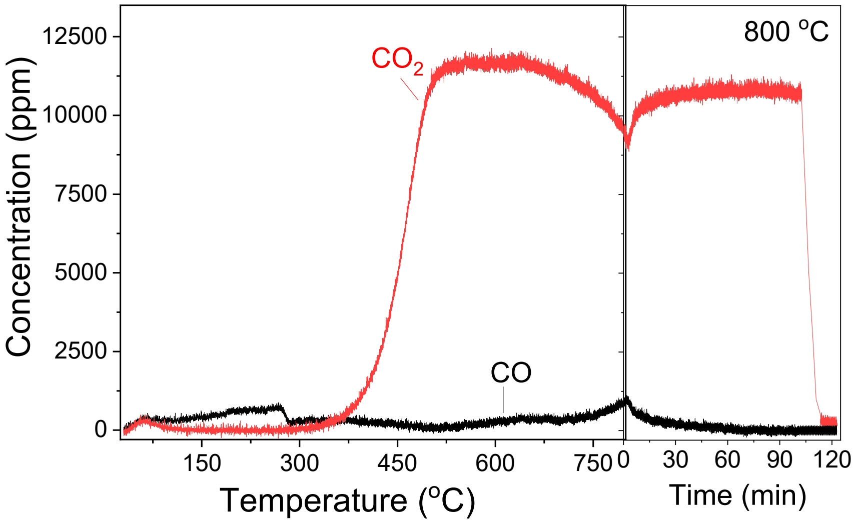

Figure S11. (a) Responses of CO_2 and CO produced during TPO experiment occurred after the TOS stability tests conducted at 650 °C presented in Fig.S6a over the 30% Ga_2O_3-Al_2O_3 catalyst. In (b), CO_2 response at 800 °C were recorded as a function of time until complete oxidation of carbon.

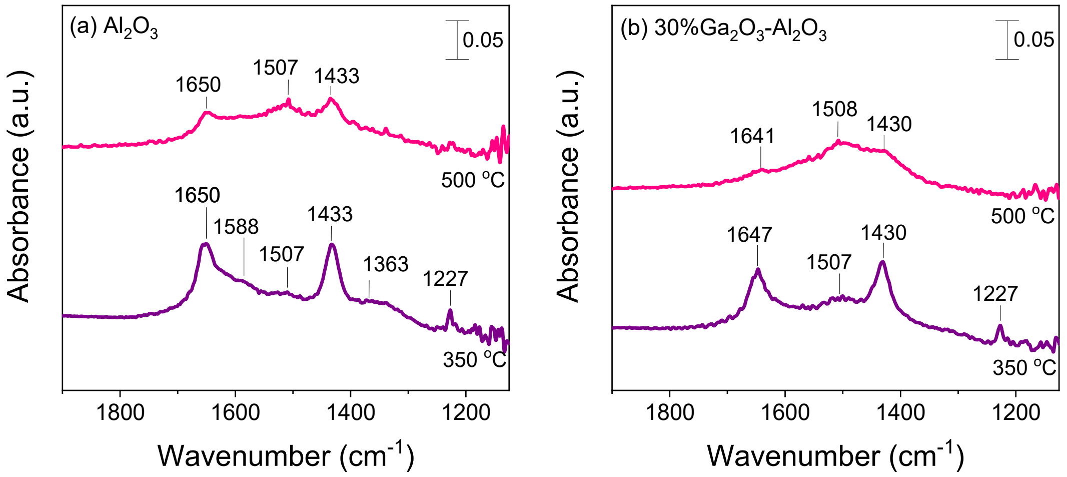

Figure S12. DRIFT spectra obtained at 350 and 500 °C in the 1900–1100 cm^{-1} region from the (a) Al_2O_3 and (b) 30%Ga_2O_3-Al_2O_3 catalysts under 1% C_3H_8 + 5% CO_2 (in He) flow.

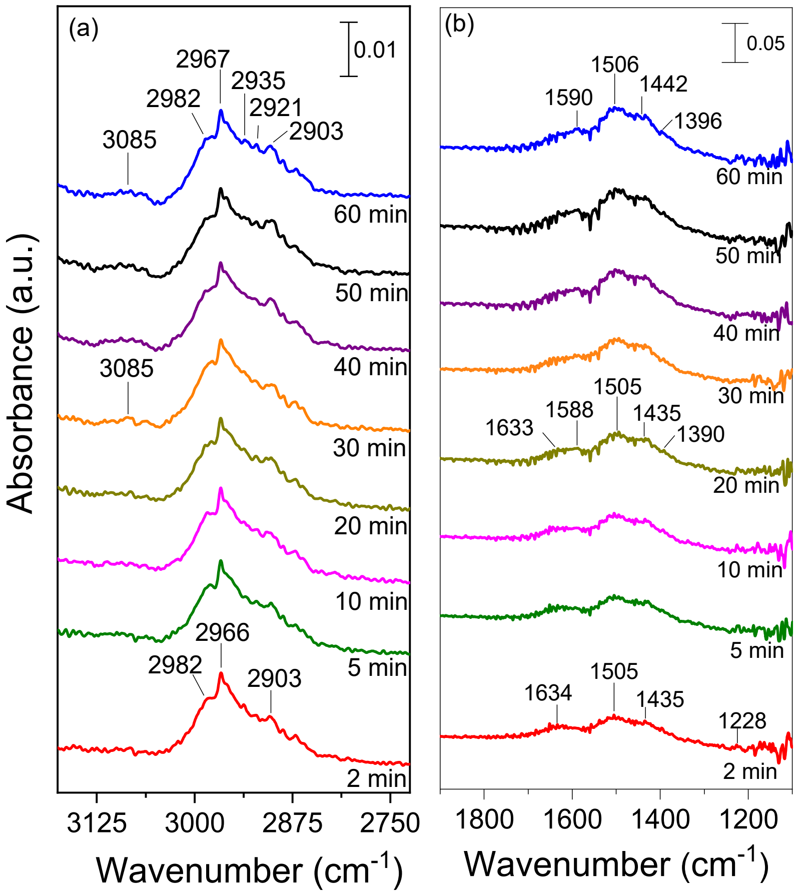

Figure S13. DRIFT spectra obtained as a function of time at 500 °C in the (a) 3175–2725 cm^{-1} and (b) 1900-1100 cm^{-1} regions following interaction of 30%Ga_2O_3-Al_2O_3 catalysts with 1% C_3H_8 + 5% CO_2 (in He).

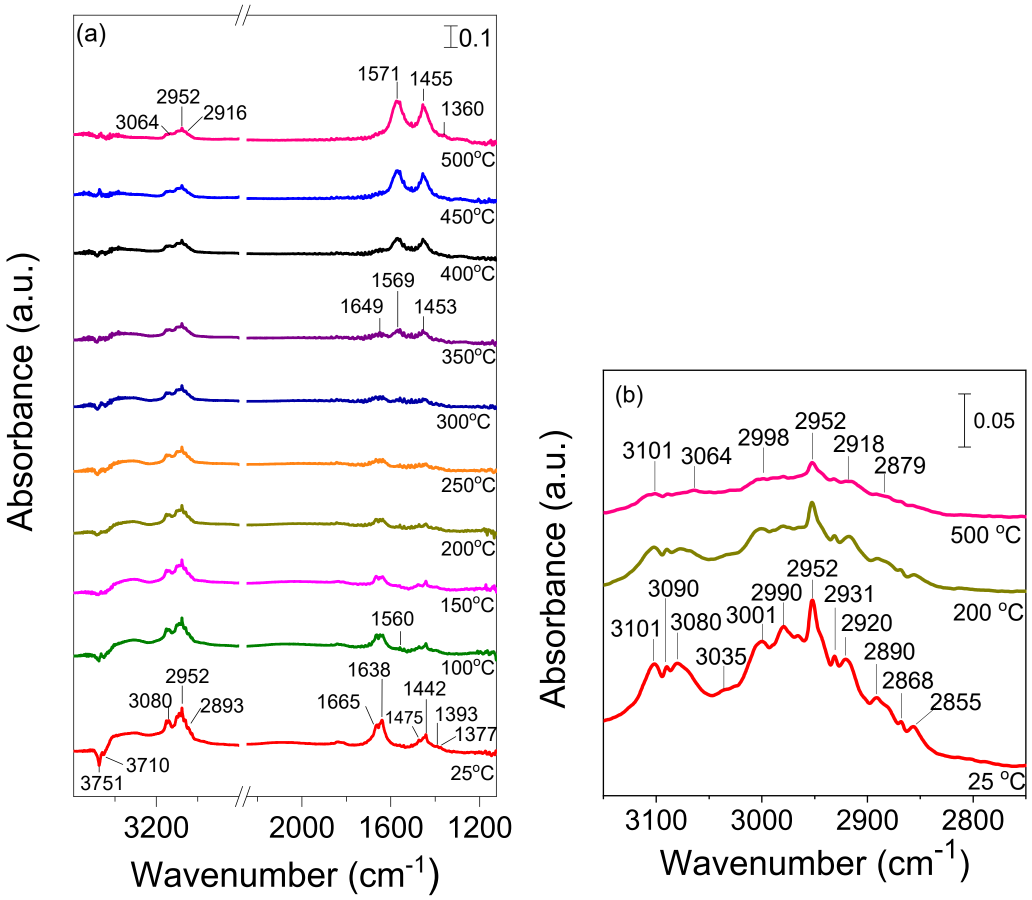

Figure S14. (a) DRIFT spectra obtained following interaction of the 30%Ga_2O_3-Al_2O_3 catalyst with 10% C_3H_6 (in He) in the temperature range of 25-500 °C. The corresponding DRIFT spectra obtained at 25, 200 and 500 °C in the 3100–2750 cm^{-1} region are presented in (b).

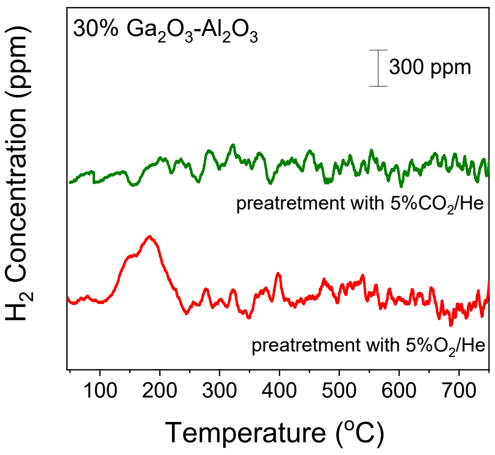

Figure S15. H_2-TPR profiles obtained from the 30%Ga_2O_3-Al_2O_3 catalyst after pre-oxidation with 5%O_2/He and 5%CO_2/He.
